# Metabolites Associated with Vigor to Frailty Among Community-Dwelling Older Black Men

**DOI:** 10.3390/metabo9050083

**Published:** 2019-04-30

**Authors:** Megan M. Marron, Tamara B. Harris, Robert M. Boudreau, Clary B. Clish, Steven C. Moore, Rachel A. Murphy, Venkatesh L. Murthy, Jason L. Sanders, Ravi V. Shah, George C. Tseng, Stacy G. Wendell, Joseph M. Zmuda, Anne B. Newman

**Affiliations:** 1 Department of Epidemiology, University of Pittsburgh, Pittsburgh, PA 15213, USA; mmm133@pitt.edu (M.M.M.); BoudreauR@edc.pitt.edu (R.M.B.); ZmudaJ@edc.pitt.edu (J.M.Z.); 2 Laboratory of Epidemiology and Population Sciences, Intramural Research Program, National Institute on Aging, Bethesda, MD 20814, USA; harris99@nia.nih.gov; 3 Broad Institute of MIT and Harvard, Cambridge, MA 02142, USA; clary@broadinstitute.org; 4 Division of Cancer Epidemiology, and Genetics, National Cancer Institute, Rockville, MD 20850, USA; steve.moore@nih.gov; 5 Centre of Excellence in Cancer Prevention, School of Population and Public Health, University of British Columbia, Vancouver, BC V6T 1Z3, Canada; rachel.murphy@ubc.ca; 6 Department of Internal Medicine, University of Michigan at Ann Arbor, Ann Arbor, MI 48109, USA; vlmurthy@med.umich.edu; 7 Division of Pulmonary and Critical Care Medicine, Brigham and Women’s Hospital, Boston, MA 02115, USA; JSANDERS2@PARTNERS.ORG; 8 Department of Medicine, Massachusetts General Hospital, Boston, MA 02114, USA; RVSHAH@PARTNERS.ORG; 9 Departments of Biostatistics and Computational & Systems Biology, University of Pittsburgh, Pittsburgh, PA 15213, USA; ctseng@pitt.edu; 10 Department of Human Genetics, University of Pittsburgh, Pittsburgh, PA 15213, USA; 11 Departments of Pharmacology and Chemical Biology and Clinical and Translational Science, University of Pittsburgh, Pittsburgh, PA 15213, USA; gstacy@pitt.edu; 12 Departments of Medicine and Clinical and Translational Science, University of Pittsburgh, Pittsburgh, PA 15213, USA

**Keywords:** metabolomics, frailty, healthy aging, African Americans

## Abstract

Black versus white older Americans are more likely to experience frailty, a condition associated with adverse health outcomes. To reduce racial disparities in health, a complete understanding of the pathophysiology of frailty is needed. Metabolomics may further our understanding by characterizing differences in the body during a vigorous versus frail state. We sought to identify metabolites and biological pathways associated with vigor to frailty among 287 black men ages 70–81 from the Health, Aging, and Body Composition study. Using liquid chromatography-mass spectrometry, 350 metabolites were measured in overnight-fasting plasma. The Scale of Aging Vigor in Epidemiology (SAVE) measured vigor to frailty based on weight change, strength, energy, gait speed, and physical activity. Thirty-seven metabolites correlated with SAVE scores (*p* < 0.05), while adjusting for age and site. Fourteen metabolites remained significant after multiple comparisons adjustment (false discovery rate < 0.30). Lower values of tryptophan, methionine, tyrosine, asparagine, C14:0 sphingomyelin, and 1-methylnicotinamide, and higher values of glucoronate, N-carbamoyl-beta-alanine, isocitrate, creatinine, C4-OH carnitine, cystathionine, hydroxyphenylacetate, and putrescine were associated with frailer SAVE scores. Pathway analyses identified nitrogen metabolism, aminoacyl-tRNA biosynthesis, and the citric acid cycle. Future studies need to confirm these SAVE-associated metabolites and pathways that may indicate novel mechanisms involved in the frailty syndrome.

## 1. Introduction

In the United States, black compared to white older adults are more likely to be frail [1]. In fact, at every age group, older community-dwelling (i.e., not in assisted living/nursing homes) black men and women had a higher prevalence of frailty than white men and women, respectively [2]. Frailty is a major public health issue due to it being a risk factor for multiple adverse health outcomes, such as falls, hospitalization, and mortality [2]. Currently, there is no gold standard to measure frailty [3]. Existing measurements are typically based on signs and symptoms that capture the ailing extreme of the distribution [2,4], but fail to simultaneously identify vigorous older adults. A sharply dichotomous or categorical classification weighted toward late stage frailty hinders identification of mechanisms at work in earlier stage frailty, which is likely the more fruitful stage for designing interventions to ameliorate later decline. Therefore, it is not surprising that the components thought to make up the frailty syndrome have not been tightly linked to underlying mechanisms.

To reduce racial disparities in health and further the understanding of the biology and pathophysiology of frailty, a deeper characterization of frailty is needed. One way to do this is through identifying metabolites associated with the full spectrum of healthy aging from vigorous to frail. Metabolites are small molecules in cells, tissues, and bodily fluids that are intermediates or end-products of metabolism (e.g., lipids, amino acids, organic acids). Metabolomics, the large-scale study of metabolites, is the closest –omics approach to the phenotype and thus, may be particularly promising since it may provide information that is more biologically coupled to the phenotype of interest. Using metabolites to identify differences in the body during a frail state may reveal new insights into altered biological processes that adapt to maintain homeostasis in the presence of evolving frailty.

The Fried frailty phenotype is a popular method of measuring frailty and is based on weight loss, self-reported exhaustion, slow gait speed, weak grip strength, and low physical activity [2]. However, this index only captures the frail extreme among older adults. To address this issue, the Scale of Aging Vigor in Epidemiology (SAVE) was developed by modifying the scoring of the same five items used in the Fried frailty phenotype, to allow for measuring both the healthy (i.e., vigorous), in addition to the unhealthy (i.e., frail), extremes [5,6]. Examining a more complete spectrum of health, from vigorous to frail, using the SAVE allows for more variation to identify correlates of healthy aging. A pilot study measured metabolites and the items used to calculate the SAVE in a subset of randomly selected black men from the Health, Aging, and Body Composition (Health ABC) study [7]. Such a well-characterized cohort allows for identifying metabolites associated with vigor to frailty, while controlling for important confounders. Therefore, the aim of this report was to identify novel metabolites and metabolic pathways associated with vigor to frailty measured using the SAVE among older community-dwelling black men.

## 2. Results

### 2.1. Characteristics of 287 Health ABC Black Men by Tertiles of SAVE Scores

Participants were 74.6 years old, on average. There was no difference in daily calories, protein intake, or body composition by SAVE tertiles (Table 1). Median levels of markers of inflammation and kidney disease were slightly higher among frailer participants. The frailest individuals had the highest prevalence of cardiovascular disease, diabetes, and pulmonary disease, as well as were taking a larger number of prescription medications, specifically medications for hypertension, diabetes, and pulmonary diseases.

### 2.2. Metabolites Correlated with SAVE scores

Among 334 metabolites (Table S1), 37 metabolites correlated with SAVE scores (*p* < 0.05) adjusting for age and study site (Table 2), most of the associations did not appear to be driven by a single item used to calculate SAVE scores. However, only 2 of the 37 metabolites were correlated with weight change (*p* < 0.05), whereas more than a third were correlated with gait speed and physical activity. Among the 37 metabolites, 14 remained significant after multiple comparisons adjustment (false discovery rate < 0.30). Eight metabolites (glucoronate, N-carbamoyl-beta-alanine, isocitrate, creatinine, C4-OH carnitine, cystathionine, hydroxyphenylacetate, and putrescine) were positively correlated with SAVE scores, indicating lower metabolite values associated with vigor and higher values associated with frailty. The remaining six metabolites (tryptophan, methionine, tyrosine, C14:0 SM, 1-methylnicotinamide, asparagine) were negatively correlated with SAVE scores, indicating higher metabolite values associated with vigor and lower values associated with frailty.

### 2.3. Attenuation of the Association between Metabolites and SAVE Scores after Additional Adjustments

**Figure 1:**
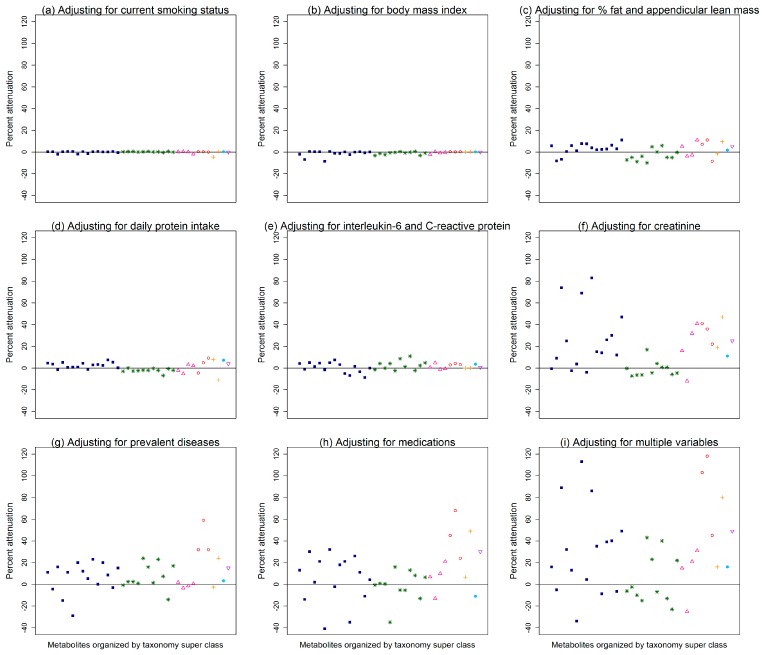
Percent attenuations of the age- and study side- adjusted correlation between 37 metabolites (organized by Human Metabolome Database taxonomy super class) and scores on the Scale of Aging Vigor in Epidemiology (SAVE) after further adjusting for more commonly measured variables: (**a**) smoking status, (**b**) body mass index, (**c**) percent body fat and appendicular lean mass, (**d**) daily protein intake, (**e**) interleukin-6 and C-reactive protein, (**f**) creatinine, (**g**) prevalent diseases (cardiovascular diseases, diabetes, and pulmonary diseases), (**h**) medications (total number of prescription medications, anti-hypertensives, and medications for diabetes), or (**i**) multiple variables (smoking status, body mass index, appendicular lean mass, percent body fat, daily protein intake, interleukin-6, C-reactive protein, creatinine, cardiovascular disease, diabetes, pulmonary diseases, and total number of prescription medications).

Figure 1 illustrates the percent attenuations of the correlations between SAVE scores and the 37 top metabolites after further adjusting for more commonly measured variables, in addition to age and study site, where each data point on the plot is a different metabolite organized by its taxonomy super class according to the Human Metabolome Database [8] (see Table S2 for the exact percent attenuation for each metabolite). Adjusting for current smoking status or body mass index minimally attenuated the associations between SAVE scores and metabolites (Figure 1a,b; attenuations ≤ 0.5%; Table S2). Adjusting for percent fat and appendicular lean mass, daily protein intake, or inflammation markers attenuated more of the associations between SAVE scores and metabolites (Figure 1c–e), though attenuations were still <12% (Table S2). Among the more commonly measured variables that were considered, adjusting for creatinine, prevalent diseases, or medications resulted in the most attenuation between SAVE scores and metabolites (Figure 1f–h; attenuations <84%, <59%, and <69%, respectively; Table S2). Interestingly, serum creatinine measured at year 1 and creatinine measured from plasma using metabolomics were highly correlated, with a correlation coefficient of 0.82 (*p* < 0.0001). Adjusting for multiple more commonly measured variables (Figure 1i) attenuated the associations between SAVE scores and 10 metabolites by more than 40%: salicylurate, 5-aminolevulinic acid, hydroxyphenylacetate, creatinine, symmetric dimethylarginine, trimethylamine-N-oxide, 2-hydroxyglutarate, glucoronate, homogentisate, and C36:4 PE (Table 2). Conversely, adjusting for multiple more commonly measured variables did not explain the association between SAVE scores and 13 metabolites (attenuations < 10%; Table 2). Specifically, the associations between SAVE scores and leucine, 1-methylnicotinamide, C54:10 triacylglycerol, glycodeoxycholate, and C22:0 sphingomyelin were actually strengthened by ≥10%, after further adjusting for multiple more commonly measured variables (Table 2).

### 2.4. Pathway Analysis

Among the 37 metabolites correlated with SAVE scores at a *p* < 0.05, 35 were in the Human Metabolome Database Version 4.0 [8] and were included in the pathway analysis. Table 3 includes the top ten pathways among 36 that involved at least one SAVE-associated metabolite. The most significant pathways were nitrogen metabolism, aminoacyl-transfer RNA biosynthesis, and the citric acid cycle. The match status for nitrogen metabolism was 5/39, meaning 39 known metabolites are involved in nitrogen metabolism and five of them were associated with SAVE scores (tyrosine, tryptophan, asparagine, histidine, and cystathionine). The match status was 6/75 for aminoacyl-transfer RNA biosynthesis (tyrosine, tryptophan, asparagine, histidine, methionine, and leucine) and 3/20 for the citric acid cycle (isocitrate, malate, and fumarate). However, low impact scores were observed for the metabolites associated with SAVE scores and involved in nitrogen metabolism, aminoacyl-transfer RNA biosynthesis, or the citric acid cycle (Table 3).

## 3. Discussion

We identified unique patterns of plasma metabolites differing across the range of health from vigorous to frail older black men. Thirty-seven metabolites correlated with SAVE scores, of which 14 remained significant after multiple comparisons adjustment. Nitrogen metabolism, aminoacyl-transfer RNA biosynthesis, and the citric acid cycle were top metabolic pathways associated with SAVE scores, suggesting differences in functioning of these pathways may be present during a vigorous versus frail state. Since many other factors influence metabolism, it was notable that several metabolites were associated with SAVE scores independent of potential confounders or mediators, such as body composition, smoking status, daily protein intake, inflammation markers, several chronic conditions, and medication use.

Several amino acids were associated with SAVE scores, indicating lower values correlated with frailer scores. Lower values of tryptophan, methionine, tyrosine, and leucine also correlated with less appendicular lean mass among the Health ABC black men [7] and lower values of leucine and other branched-chain amino acid-related metabolites correlated with lower thigh muscle cross-sectional area and fat-free mass index among functionally-limited older adults [9]. To date, few studies examined metabolites associated with frailty. A targeted set of metabolites in muscle biopsies similarly found tryptophan, methionine, tyrosine, asparagine, and histidine lower among frail older adults [10]. In addition, blood-based tryptophan and tyrosine measured among a Spanish older adult cohort were lower among frail participants [11]. Conversely, higher levels of amino acids have been associated with obesity, diabetes, and cardiovascular disease [12,13,14]. Notably, frailer Health ABC black men were more likely to have diabetes, though there was no difference in body mass index by level of frailty. It may be that the difference in direction of associations between amino acids and adverse health outcomes may be explained by a U-shaped relationship, where higher values of certain amino acids are associated with metabolic disorders, such as obesity, diabetes, and cardiovascular disease, but lower values are associated with wasting disorders that are further along in pathogenesis, such as frailty.

It was previously reported that seven metabolites (glucoronate, tryptophan, asparagine, C24:1 ceramide (d18:1), 2-hydroxyglutarate, salicylurate, and C54:10 triacylglycerol) correlated with gait speed among the Health ABC black men [15], of which all were similarly associated with SAVE scores, as expected since gait speed is an item of the SAVE. In addition, eight metabolites (N-carbamoyl-beta-alanine, creatinine, C4-OH carnitine, 5-aminolevulinic acid, inosine, symmetric dimethylarginine, C36:4 PE, and C18:2 CE) that predicted incident disability [15] and nine metabolites (glucoronate, N-carbamoyl-beta-alanine, isocitrate, creatinine, hydroxyphenylacetate, 5-aminolevulinic acid, symmetric dimethylarginine, urate, and trimethylamine-N-oxide) that were associated with extremes of a healthy aging index [16] among the Health ABC black men were also associated with SAVE scores. N-carbamoyl-beta-alanine, creatinine, inosine, and symmetric dimethylarginine are indicators of kidney functioning [15], and may be important markers of healthy aging.

SAVE-associated metabolites involved in nitrogen metabolism and aminoacyl-transfer RNA biosynthesis were mostly amino acids. In a healthy individual, plasma levels of amino acid are tightly regulated within a fixed range [17,18]. The rate of appearance of amino acids in plasma is a result of dietary protein intake and release of amino acids by muscles and other tissues, whereas the rate of disappearance from plasma is due to amino acid oxidation, metabolism, and incorporation into proteins, and, to a lesser degree, loss through excretion. Hypoaminoacidemia may occur from insufficient protein intake/storage and/or stress [17]. Protein intake in Health ABC did not vary by level of frailty and appeared to be sufficient with an overall average of 0.97 g/kg/day. However, it is possible that this level of intake is insufficient in the frailer men to overcome aging-related anabolic resistance, where the body’s ability to use amino acids to synthesize muscle proteins appears to be altered [19,20,21]. Additionally, or alternatively, the frailer participants may have lower levels of tryptophan, methionine, tyrosine, asparagine, and histidine due to an increased stress response causing conversion of plasma amino acids to glucose [17]. There may be specific mechanisms that adapt to aging-related disease states, but by doing so have adverse effects that potentially lead to altered energy pathways and then eventually frailty.

It should be noted that when examining associations between the 37 SAVE-associated metabolites and the individual components of the SAVE, only two metabolites were correlated with weight change, whereas more than a third of the metabolites were correlated with gait speed and physical activity. A limitation of this report was that measurements for metabolites were unit-less liquid chromatography-mass spectrometry (LC-MS) peak areas. If we instead had concentrations of metabolites, we could determine whether the metabolites that were either lower or higher among frailer participants were more extreme than what is considered within a healthy range. In addition, we measured frailty severity using the SAVE, which only described how frail an individual was relative to the rest of their cohort, whereas that same individual may appear much less frail if they were instead compared to the United States population of older adults. Other limitations include studying only black men, limiting the generalizability and comparability of results; using self-reported dietary information from the Food Frequency Questionnaire, which may not be accurate to what the participants were actually consuming, as well as it only provides information on usual diet; and the potential for false positives given the liberal false discovery rate. Strengths were the well-characterized cohort of ambulatory older adults, allowing us to examine whether several potential factors attenuated the associations between metabolites and SAVE scores, as well as information on a large number of metabolites from plasma samples carefully collected and stored after an overnight fast.

Several metabolites, particularly amino acids, were associated with vigor to frailty scores among older black men from the Health ABC study, which may help us better understand the mechanisms underlying progression of frailty. Multisystem decline with frailty makes it impossible to pinpoint any one organ system responsible; instead the aggregate of multisystem dysfunction may actually be responsible for these metabolic characteristics. The generalizability of these findings in the Health ABC black men needs to be confirmed. Once confirmed, more research will be needed to identify the biologic mechanisms causing these differences in metabolites that are associated with frailty, either through animal models that can directly alter specific pathways or through interventions in humans that attempt to enhance specific pathways.

## 4. Materials and Methods

### 4.1. The Health, Aging, and Body Composition (Health ABC) Study

The Health ABC study was a prospective cohort of 3075 black and white men and women recruited from Pittsburgh, Pennsylvania and Memphis, Tennessee during March 1997 to July 1998. The study was originally designed to address the role of weight-related health conditions and body composition in the onset of disability [22]. Eligible participants were ages 70–79 during recruitment and self-reported no difficulty walking ¼ mile, climbing ten steps, or with basic activities of daily living. Ineligibility included history of active cancer treatment in the past three years or planning on moving from the study area within the next three years. The study was approved by each site’s institutional review board. Participants provided written informed consent.

An ancillary pilot study measured 350 known and numerous unknown metabolites in a randomly selected subset of 319 black men from the second visit (year 2) of the Health ABC study to provide insight on the influence of lean mass and adiposity in human metabolism [7]. The study was limited in size, so it was restricted to black men since there was a higher prevalence of obesity and obesity-related health conditions, but more muscle mass among black versus white Americans, and to limit heterogeneity due to differences in body composition by sex. The randomly selected black men were healthier than the whole sample of Health ABC black men since the second visit was used and attrition had occurred during the first year [7].

### 4.2. Metabolites

Metabolites were measured in plasma extracts collected at Visit 2 in the morning after overnight fasting for at least eight hours (mean = 14 h). We used plasma samples that had never been thawed and were stored at −80 °C from the time of collection (1998–99) until 2016 when metabolites were measured. Using LC-MS, metabolite profiling platforms (Table S3) measured: (1) amines and polar metabolites (e.g., amino acids, dipeptides), (2) central metabolites and polar metabolites (e.g., sugars, organic acids, purine and pyrimidines), and (3) lipids (e.g., triglycerides). All profiling methods were not of a targeted nature. Metabolite values used for this report are LC-MS peak areas, analyzed using TraceFinder (ThermoFisher Scientific, Waltham, MA, USA) and Progenesis QI (Nonlinear Dynamics, UK). Peaks were confirmed manually using known standards. Metabolites below the limit of quantitation (signal/noise < 10) were classified as unquantifiable [23]. The median intraclass correlation coefficient of known metabolites from 16 blinded duplicates was 0.92 (interquartile range: 0.81–0.97), indicating high reliability [7]. Two pooled samples were run after every 20 study-samples. One pool was used to normalize the data, if necessary, and the second pool was used to assess quality of that normalization. Data from the positive and negative ion mode MS detection was normalized to the nearest neighbor and data from the lipid profiling method was not normalized.

Positive ion mode detection used a 4000 QTRAP triple quadrupole mass spectrometer (SCIEX, Framingham, MA, USA) coupled to an 1100 Series pump (Agilent, Santa Clara, CA, USA) and an HTS PAL autosampler (Leap Technologies, Morrisville, NC, USA) with a 4.5 kV ion spray voltage and at 450 °C source temperature. Using protein precipitation, plasma samples (10 µL) were prepared with the addition of nine volumes of 74.9:24.9:0.2 (*v*/*v*/*v*) acetonitrile/methanol/formic acid containing stable isotope-labeled internal standards (0.2 ng/μL valine-d_8_, Isotec; and 0.2 ng/μL phenylalanine-d_8_; Cambridge Isotope Laboratories). Samples were centrifuged for 10 min (9000× *g*, 4 °C). Resulting supernatants were injected onto a 150 × 2 mm Atlantis HILIC column that was eluted at a 250 µL/min flow rate. Initial conditions were set at 5% mobile phase A (10mM ammonium formate and 0.1% formic acid in water) for one minute and then altered linearly over ten minutes to 40% mobile phase B (acetonitrile with 0.1% formic acid) [23,24].

Negative ion mode detection used a 5500 QTRAP triple quadrupole mass spectrometer (SCIEX) coupled to an ACQUITY UPLC (Waters, Milford, MA, USA) with a modified hydrophilic interaction chromatography method and −4.5kV ion spray voltage and at 500 °C source temperature. Using protein precipitation, plasma samples (30 µL) were prepared with the addition of 120 μL of 80% methanol containing 0.05 ng/μL [^15^N_4_]-inosine, 0.05 ng/μL thymine-d_4_, and 0.1ng/μL glycocholate-d_4_ as internal standards. Samples were centrifuged (10 min, 9000× *g*, 4 °C) and 10 µL of supernatants were injected onto a 150 × 2.0 mm Luna NH_2_ column (Phenomenex) that underwent elution at a 400 µL/min flow rate. Initial conditions were set at 10% mobile phase A (20 mM ammonium acetate and 20 mM ammonium hydroxide; Sigma-Aldrich) in water (VMR) along with 90% mobile phase B (10 mM ammonium hydroxide in 75:25 *v*/*v* acetonitrile/methanol (VWR)) and then altered linearly over ten minutes to 100% mobile phase A [23,24].

Lipids were detected using an Exactive Plus orbitrap mass spectrometer (Thermo Fisher Scientific, Waltham, MA, USA) coupled to a Nexera X2 UHPLC (Shimadzu, Marlborough, MA, USA) with electrospray ionization and positive ion mode Q1 scans. The ion spray voltage was 5.0 kV with 400 °C source temperature. Plasma samples (10 µL) were extracted using 190 μL of isopropanol containing 0.25 ng/μL 1-dodecanoyl-2-tridecanoyl-sn-glycero-3-phosphocholine (Avanti Polar Lipids). Samples were centrifuged and 10 µL of supernatants were injected onto a 150 × 3.0 mm Prosphere HP C4 column (Grace). The column was eluted with initial conditions set at 80% mobile phase A (95:5:0.1 vol/vol/vol 10 mM ammonium acetate/methanol/acetic acid), then after two minutes, changed linearly over one minute to 80% mobile phase B (99.9:0.1 vol/vol methanol/acetic acid), followed by a linear change over 12 min to 100% mobile phase B. Conditions remained at 100% mobile phase B for 10 min [23,24].

### 4.3. Scale of Aging Vigor in Epidemiology (SAVE)

The SAVE was developed by modifying the Fried frailty phenotype [2] to allow for measuring both the healthy (i.e., vigorous), in addition to the unhealthy (i.e., frail), extremes [5,6]. The SAVE was calculated using information from five items assessed at Visit 2: weight change, physical activity, grip strength, gait speed, and energy level. Weight change was the difference between measurements at Visits 1 and 2. Self-reported time spent doing major chores, walking, climbing stairs, working, volunteering, and caregiving in the past week was converted to kilocalories/kilogram/week and summed to get an estimate of weekly physical activity. Grip strength was the maximum of two trials on the right hand using a hand-held dynamometer. Gait speed was the average over 20 m. Participants self-reported usual energy level in the past month on a scale of 0 (no energy) to 10 (most energy ever had). Scores on each of the five items were ranked into tertiles using information from all Health ABC men (Table 4). Individuals who scored in the best, middle, or worst tertile for a component received a score of 0, 1, or 2, respectively. SAVE scores were the sum of tertile scores for the five items, ranging from 0 (most vigorous) to 10 (most frail), and examined continuously and as tertiles. SAVE tertiles were determined using information from all Health ABC participants and ranged from 0–3 (most vigorous), 4–5, and 6–10 (most frail).

#### Health ABC Black Men with Information on Metabolites and the SAVE

Among the 319 black men with metabolites measured, 287 (90%) had complete information to calculate the SAVE. In this report, we focused on the known metabolites. Among the 350 known metabolites, 301 were measured in all 287 participants and 33 were measured in at least 80% of participants, of which missing values were assumed to be due to the true values being below the detectable limit and were replaced with half the minimum recorded value for that respective metabolite [7]. Sixteen (5%) metabolites were excluded from the current analysis because they were measured in less than 80% of participants [25]. Therefore, we examined 334 metabolites among 287 black men (Table S1). Table S4 includes the 334 metabolites organized by taxonomy class according to the Human Metabolome Database [8].

### 4.4. Potential Confounders or Mediators of Metabolites and SAVE Scores

Participants self-reported age, race, highest level of education, and smoking habits at baseline (i.e., year 1). Height and weight were recorded at Visit 2 (i.e., year 2). Baseline history or presence of cardiovascular disease, hypertension, diabetes, cancer, peripheral arterial disease, osteoarthritis, depression, pulmonary disease, and kidney disease were based on self-report of a physician diagnosis. Participants were also classified as having cardiovascular disease, hypertension, diabetes, cancer, depression, or pulmonary disease if taking medication for those diseases and peripheral arterial disease if self-reported intermittent claudication, leg pain, or leg artery bypass or angioplasty. Participants brought all prescription medications used in the last two weeks to Visit 2 for a medication inventory.

Daily calories, protein, and fat intake at Visit 2 were determined using a 108-item interviewer-administered food frequency questionnaire estimating usual nutrient intake over the past year and was developed for the Health ABC study by Block Dietary Data Systems (Berkeley, CA) using food lists obtained from a 24 h recall among participants who were ages 65 or older, black or white race, and living in the Northeastern or Southern United States from the Third National Health and Nutrition Examination Survey [26]. Protein per kilogram of body weight and percent of kilocalories from protein and from fat were also examined.

Body composition at Visit 2 was estimated using total body dual-energy x-ray absorptiometry (Hologic QDR 4500A; Hologic, Bedford, MA). Appendicular lean mass was the bone-free lean mass in the arms and legs standardized to height^2^. Percent fat was examined relative to total body mass.

A core laboratory at Wake Forest University measured interleukin-6 and C-reactive protein in serum and EDTA-plasma, respectively, collected at Visit 2 in the morning after an overnight fast. Cystatin C and creatinine were measured in serum at baseline by the Laboratory for Clinical Biochemistry Research at the University of Vermont. Glomerular filtration rate was estimated as 133*(cystatin C/0.8)^−y^*0.996^age^, where y = 0.499 when cystatin C ≤ 0.8 mg/L and y = 1.328 when cystatin C > 0.8 mg/L [27].

### 4.5. Statistical Analysis

Mean (standard deviation) or frequency (percent) described differences in potential confounders or mediators by SAVE tertiles and were tested using Analysis of Variance or Kruskal–Wallis for continuous measures and chi-square tests or Fisher’s exact test for categorical measures. Metabolites were log-transformed and standardized. Using SAS 9.4, partial Pearson correlation coefficients identified metabolites associated with SAVE scores, while minimally adjusting for age and study site. A Benjamini–Hochberg correction was used to account for multiple comparisons [28]. Since this was a hypothesis-generating report, we used a liberal 30% false discovery rate [25]. The associations between metabolites and SAVE scores were initially adjusted for only age and study site because we wanted to first identify *all* metabolites related to SAVE scores, not just metabolites associated above and beyond certain risk factors. After identifying metabolites associated with SAVE scores, we then determined whether associations were attenuated after further adjusting for more commonly measured variables. We examined the extent to which more commonly measured variables explained the age- and study site-adjusted associations between metabolites and SAVE scores using percent attenuation calculated as 100*(r_1_ − r_2_)/r_1_, where r_1_ is the age- and study site-adjusted correlation coefficient between SAVE scores and metabolite values and r_2_ is the correlation coefficient after further adjusting for more commonly measured variables.

Metabolites associated with SAVE scores at a *p* < 0.05 were examined in a pathway analysis using MetaboAnalyst [29], which compared the set of associated metabolites against established sets of metabolites involved in metabolic pathways. A Fisher’s exact test determined whether the number of SAVE-associated metabolites involved in a pathway was more than expected by chance. Impact scores indicated how centrally located SAVE-associated metabolites were in particular pathways, i.e., the amount of impact on the pathway if the values for those metabolites were altered. Impact scores range from zero to one, indicating matched metabolites account for none to all of the pathway importance, respectively [29].

## Figures and Tables

**Table 1:** Characteristics of 287 black men from the Health, Aging, and Body Composition study by tertiles of scores on the Scale of Aging Vigor in Epidemiology (SAVE).

Mean (Standard Deviation) or Frequency (Percent)	SAVE Tertiles			Overall *p*-Value, Pairwise Comparisons
	Vigorous (T1) *n* = 73	Average (T2) *n* = 105	Frail (T3) *n* =1 09	
SAVE scores	2.4 (0.7) Range: 0–3	4.5 (0.5) Range: 4–5	7.0 (1.1) Range: 6–10	-
Age	74 (3)	75 (3)	75 (3)	0.006, T1 < T2, T3
Pittsburgh site	34 (47%)	56 (53%)	63 (58%)	0.33
More than high school education	28 (38%)	24 (23%)	28 (26%)	0.06
Current smoker at baseline	9 (12%)	22 (21%)	21 (19%)	0.31
Body mass index (kg/m^2^)	27 (4)	27 (4)	27 (5)	0.82
Dietary intake:				
Total calories (Kcal/day)	2329 (1111)	2199 (1022)	2095 (1038)	0.35
Protein intake (g/day)	81 (44)	75 (37)	73 (39)	0.41
Percent of daily calories from protein	14 (3)	14 (3)	14 (3)	0.82
Daily protein intake per body weight (g/kg)	1.0 (0.6)	0.97 (0.5)	0.94 (0.5)	0.71
Fat intake (g/day)	92 (51)	87 (49)	81 (48)	0.30
Percent of daily calories from fat	35 (6)	35 (8)	34 (8)	0.57
Body composition:				
Appendicular lean mass (kg/m^2^)	8.4 (1)	8.3 (1)	8.3 (1)	0.68
Percent fat	28 (5)	28 (5)	28 (6)	0.92
Inflammation markers:				
Interleukin-6 (pg/mL)	4.2 (5.9) Median = 2.5	3.2 (2.2) Median = 2.4	4.2 (3.4) Median = 3.0	0.05
C-reactive protein (ug/mL)	5.4 (8.9) Median = 2.8	5.3 (9.7) Median = 2.1	8.4 (16) Median = 3.9	0.05
Markers of kidney disease at baseline:				
Creatinine (mg/dL)	1.2 (0.2) Median = 1.2	1.2 (0.3) Median = 1.2	1.3 (0.4) Median = 1.2	0.04
Cystatin C (mg/L)	1.0 (0.2) Median = 0.96	1.0 (0.3) Median = 1.0	1.1 (0.3) Median = 1.1	0.05
Glomerular filtration rate	77 (17)	75 (19)	70 (19)	0.03, T1 > T3
Prevalent disease at baseline:				
Cardiovascular disease	11 (15%)	36 (34%)	39 (36%)	0.006, T1 < T2, T3
Hypertension	34 (47%)	65 (62%)	67 (61%)	0.08
Diabetes	8 (11%)	18 (17%)	37 (34%)	0.0004, T1, T2 < T3
Cancer	10 (14%)	11 (10%)	11 (10%)	0.72
Peripheral artery disease	2 (3%)	7 (7%)	9 (8%)	0.32
Osteoarthritis	2 (3%)	9 (9%)	11 (10%)	0.17
Depression	4 (5%)	5 (5%)	8 (7%)	0.71
Pulmonary disease	7 (10%)	8 (8%)	21 (19%)	0.02, T2<T3
Kidney disease	1 (1%)	2 (2%)	0	0.36
Medication use:				
Total number of prescription medications	2.2 (2)	3.0 (3)	4.0 (4)	0.0003, T1,T2 < T3
Antihypertensive medications	35 (48%)	64 (61%)	74 (68%)	0.03, T1 < T3
Antilipemic medications	14 (19%)	11 (10%)	17 (16%)	0.25
Medications for diabetes:	5 (7%)	17 (16%)	36 (33%)	<0.0001, T1, T2 < T3
Insulin	0	2 (2%)	10 (9%)	0.004, T1, T2 < T3
Oral hypoglycemic	5 (7%)	15 (14%)	28 (26%)	0.003, T1, T2 < T3
Medications for prostate disease	10 (14%)	14 (13%)	19 (17%)	0.83, P = 0.66
Medications for pulmonary diseases	5 (7%)	2 (2%)	13 (12%)	0.02, T2 < T3
Spasmolytics (theophylline and others)	0	1 (1%)	5 (5%)	0.09
Anti-inflammatory	24 (33%)	43 (41%)	52 (49%)	0.09

**Table 2:** Age and study site-adjusted correlation of 37 top metabolites with scores on the Scale of Aging Vigor in Epidemiology (SAVE) (*p* < 0.05) and attenuations after further adjustments among 287 black men from the Health, Aging, and Body Composition study.

Log-Transformed and Standardized Metabolites	Human Metabolome Database ID Number	Human Metabolome Database Taxonomy Sub Class	Continuous SAVE Scores, Adjusting for Age and Study Site (*N* = 287)		Continuous SAVE Scores, Adjusting for Multiple Variables ^1^ (*n* = 257)	
			Correlation, *p*-Value	False Discovery Rate	Correlation, *p*-Value	Percent Attenuation ^2^
Glucuronate	HMDB00127	Carbohydrates/carbohydrate conjugates	0.21, *p* = 0.0003	0.08	0.12, *p* = 0.07	49%
Tryptophan	HMDB00929	Indolyl carboxylic acids/derivatives	−0.21, *p* = 0.0005	0.08	−0.18, *p* = 0.005	15%
Methionine	HMDB00696	Amino acids/peptides/analogues	−0.19, *p* = 0.001	0.15	−0.13, *p* = 0.04	16%
N-carbamoyl-beta-alanine	HMDB00026	Ureas	0.17, *p* = 0.004	0.22	0.13, *p* = 0.045	39%
Tyrosine	HMDB00158	Amino acids/peptides/analogues	−0.17, *p* = 0.004	0.22	−0.16, *p* = 0.01	−5%
Isocitrate	HMDB00193	Tricarboxylic acids and derivatives	0.17, *p* = 0.004	0.22	0.11, *p* = 0.08	40%
Creatinine	HMDB00562	Amino acids/peptides/analogues	0.16, *p* = 0.008	0.27	0.02, *p* = 0.79	89%
C4-OH carnitine	HMDB13127	Beta hydroxy acids/derivatives	0.16, *p* = 0.009	0.27	0.10, *p* = 0.14	34%
C14:0 SM	HMDB12097	Phosphosphingolipids	−0.15, *p* = 0.009	0.27	−0.14, *p* = 0.03	−6%
Cystathionine	HMDB00099	Amino acids/peptides/analogues	0.15, *p* = 0.009	0.27	0.11, *p* = 0.09	32%
Hydroxyphenylacetate	HMDB00020	1-hydroxy-2-unsubstituted benzenoids	0.15, *p* = 0.01	0.27	−0.004, *p* = 0.95	103%
Putrescine	HMDB01414	Amines	0.15, *p* = 0.01	0.27	0.11, *p* = 0.09	16%
1-methylnicotinamide	HMDB00699	Pyridinecarboxylic acids/derivatives	−0.15, *p* = 0.01	0.27	−0.18, *p* = 0.004	−25%
Asparagine	HMDB00168	Amino acids/peptides/analogues	−0.15, *p* = 0.01	0.27	−0.12, *p* = 0.07	13%
Leucine	HMDB00687	Amino acids/peptides/analogues	−0.14, *p* = 0.02	0.35	−0.19, *p* = 0.003	−34%
5-aminolevulinic acid	HMDB01149	Amino acids/peptides/analogues	0.14, *p* = 0.02	0.36	−0.02, *p* = 0.81	113%
Inosine	HMDB00195	Not available	0.14, *p* = 0.02	0.39	0.12, *p* = 0.06	16%
Histidine	HMDB00177	Amino acids/peptides/analogues	−0.13, *p* = 0.03	0.39	−0.12, *p* = 0.07	4%
C34:3 PE plasmalogen	HMDB11343	Glycerophosphoethanolamines	−0.13, *p* = 0.03	0.39	−0.08, *p* = 0.21	−15%
Symmetric dimethylarginine (SDMA)	HMDB03334	Amino acids/peptides/analogues	0.13, *p* = 0.03	0.39	0.02, *p* = 0.77	86%
C24:1 ceramide (d18:1)	HMDB04953	Ceramides	0.13, *p* = 0.03	0.39	0.12, *p* = 0.06	−7%
C36:4 PE	HMDB08937	Glycerophosphoethanolamines	0.13, *p* = 0.03	0.39	0.07, *p* = 0.31	43%
Urate	HMDB00289	Purines/purine derivatives	0.13, *p* = 0.03	0.39	0.11, *p* = 0.09	21%
C18:2 CE	HMDB00610	Steroid esters	−0.13, *p* = 0.03	0.39	−0.09, *p* = 0.17	22%
Trimethylamine-N-oxide	HMDB00925	Aminoxides	0.13, *p* = 0.03	0.39	0.02, *p* = 0.73	80%
2-hydroxyglutarate	HMDB00694	Short-chain hydroxy acids/derivatives	0.13, *p* = 0.03	0.39	0.07, *p* = 0.29	49%
C24:0 SM	HMDB11697	Phosphosphingolipids	−0.13, *p* = 0.03	0.39	−0.13, *p* = 0.049	−3%
Fumarate	HMDB00134	Dicarboxylic acids/derivatives	0.13, *p* = 0.03	0.39	0.19, *p* = 0.002	−7%
C22:0 SM	HMDB12103	Phosphosphingolipids	−0.13, *p* = 0.03	0.39	−0.15, *p* = 0.02	−10%
C20:5 LPC	HMDB10397	Glycerophosphocholines	−0.12, *p* = 0.04	0.39	−0.06, *p* = 0.36	40%
Salicylurate	HMDB00840	Benzoic acids/derivatives	0.12, *p* = 0.04	0.41	−0.02, *p* = 0.75	118%
Homogentisate	HMDB00130	Phenylacetic acids	0.12, *p* = 0.04	0.41	0.08, *p* = 0.19	45%
Glycodeoxycholate	HMDB00631	Bile acids, alcohols and derivatives	−0.12, *p* = 0.04	0.42	−0.13, *p* = 0.045	−13%
Malate	HMDB00156	Beta hydroxy acids and derivatives	0.12, *p* = 0.04	0.42	0.16, *p* = 0.01	−9%
5-hydroxytryptophan	HMDB00472	Tryptamines and derivatives	−0.12, *p* = 0.04	0.42	−0.10, *p* = 0.13	31%
C54:10 TAG	----	Triradylcglycerols	−0.12, *p* = 0.046	0.43	−0.16, *p* = 0.01	−23%
C44:13 PE plasmalogen	----	Glycerophosphoethanolamines	−0.12, *p* = 0.049	0.44	−0.07, *p* = 0.29	23%

^1^ Current smoking status, body mass index, appendicular lean mass, percent body fat, daily protein intake, interleukin-6, C-reactive protein, creatinine, cardiovascular disease, diabetes, pulmonary diseases, and total number of prescription medications. ^2^ Percent attenuation = 100*(r_1_−r_2_)/r_1_; where r_1_ = correlation coefficient between SAVE scores and a metabolite, adjusting for age and study site, r_2_ = correlation coefficient after further adjustments. Note: 30 participants were missing appendicular lean mass, percent body fat, daily protein intake, interleukin-6, C-reactive protein, and/or creatinine, thus, the correlation coefficient that was further adjusted for those variables was compared to the age- and study site-adjusted correlation coefficient restricted to the same sample size to calculate percent attenuation. Grey shading indicates metabolites that were significant (*p* < 0.05) after adjusting for multiple more commonly measured variables. SM = sphingomyelin. PE = phosphatidylethanolamine. CE = cholesteryl ester. LPC = lysophosphatidylcholine. TAG = triacylglycerol.

**Table 3:** Top results from a pathway analysis of 35 top metabolites ^1^ correlated with scores on the Scale of Aging Vigor in Epidemiology (SAVE; *p* < 0.05) among 287 black men from the Health, Aging, and Body Composition study.

Pathways	Match Status	Fisher’s Exact Test *p*-Value	False Discovery Rate	Impact Score
Nitrogen metabolism	5/39	0.00009	0.007	0.008
Aminoacyl-tRNA biosynthesis	6/75	0.0002	0.01	0
Citric acid cycle	3/20	0.002	0.05	0.12
Tyrosine metabolism	4/76	0.013	0.27	0.15
Phenylalanine metabolism	3/45	0.02	0.28	0
Glycine, serine and threonine metabolism	3/48	0.02	0.28	0
Alanine, aspartate and glutamate metabolism	2/24	0.03	0.37	0.05
Sphingolipid metabolism	2/25	0.04	0.37	0.30
Phenylalanine, tyrosine and tryptophan biosynthesis	2/27	0.04	0.37	0.007
beta-Alanine metabolism	2/28	0.05	0.37	0.04

^1^ The following 35 out of 37 metabolites that were correlated with SAVE scores at *p* < 0.05 had an identification number in the Human Metabolome Database and were included in the pathway analysis: glucuronate, tryptophan, methionine, N-carbamoyl-beta-alanine, tyrosine, isocitrate, creatinine, C4-OH carnitine, C14:0 SM, cystathionine, hydroxyphenylacetate, putrescine, 1-methylnicotinamide, asparagine, leucine, 5-aminolevulinic acid, inosine, histidine, C34:3 PE plasmalogen, SMDA, C24:1 ceramide (d18:1), C36:4 PE, urate, C18:2 CE, trimethylamine-N-oxide, 2-hydroxyglutarate, C24:0 SM, fumarate, C22:0 SM, C20:5 LPC, salicylurate, homogentisate, glycodeoxycholate/glycochenodeoxycholate, malate, and 5-hydroxytryptophan.

**Table 4:** Tertile cut-offs of the five items used to calculate the Scale of Aging Vigor in Epidemiology (SAVE) for 287 black men from the Health, Aging, and Body Composition (Health ABC) study based on information from all Health ABC men.

Five Items Used to Calculate the SAVE:	Best Tertile = 0	Mid Tertile = 1	Worst Tertile = 2
1. Weight change (kg)	>0.68	<−1.36 to ≤0.68	≤−1.36
2. Physical activity ^1^ (kcal/kg/week)	≥43	>11 to <43	≤11
3. 20 m walk time (sec)**	≤16	>16 to ≤18	>18
4. Grip strength (kg):			
BMI < 24	>38	>32 to ≤38	≤32
BMI ≥ 24	>41	>35 to ≤41	≤35
5. Usual energy level	8 to 10	6 to 7	0 to 5

^1^ Total physical activity is based off of kcal/kg/week doing major chores, walking and climbing stairs, working, volunteering and caregiving (did not collect data for kcal/kg/week of exercise/recreation). ^2^ Tertiles for walk time did not differ for men by mean height.

## Supplementary Materials

The following are available online at https://www.mdpi.com/2218-1989/9/5/83/s1, Table S1: Correlation coefficients of SAVE scores and 334 metabolites among N = 287 Health ABC black men, adjusting for age and study site, Table S2: Attenuation of correlation between SAVE scores and top 37 metabolites after further adjustments in addition to age and study site, organized by taxonomy classification, Table S3: Metabolites detected among overnight-fasting plasma samples from 287 Health ABC black men by the three profiling platforms, Table S4: Taxonomy classification based on the Human Metabolome Database of 334 metabolites detected in overnight-fasting plasma from 287 Health ABC black men.
