# Supplementary material for: Metabolites Associated with Vigor to Frailty Among Community-Dwelling Older Black Men
Source: Metabolites. 2019 Apr 30;9(5):83. doi: 10.3390/metabo9050083 (PMC6572139; doi:10.3390/metabo9050083)
Supplement: Supplementary file 1 [file metabolites-09-00083-s001.pdf]

Metabolites associated with vigor to frailty among community-dwelling older black men
Supplemental Information

Table S1. Correlation coefficients of SAVE scores and 334 metabolites among N=287 Health ABC black men, adjusting for age and study site

Log-transformed and standardized Metabolites	Metabolite profiling method*	Human Metabolome Database ID	Human Metabolome Database Taxonomy sub class	Continuous SAVE scores		
				Partial Pearson correlation	p-value	False discovery rate
Glucoronate	HILIC-neg	HMDB00127	Carbohydrates and carbohydrate conjugates	0.21149	0.0003	0.0818
Tryptophan	HILIC-pos	HMDB00929	Indolyl carboxylic acids and derivatives	-0.2052	0.0005	0.0818
Methionine	HILIC-pos	HMDB00696	Amino acids, peptides, and analogues	-0.18887	0.0014	0.1512
N-carbamoyl-beta-alanine	HILIC-pos	HMDB00026	Ureas	0.17218	0.0035	0.2195
Tyrosine	HILIC-pos	HMDB00158	Amino acids, peptides, and analogues	-0.17156	0.0037	0.2195
Isocitrate	HILIC-neg	HMDB00193	Tricarboxylic acids and derivatives	0.17026	0.0039	0.2195
Creatinine	HILIC-pos	HMDB00562	Amino acids, peptides, and analogues	0.15638	0.0082	0.2749
C4-OH carnitine	HILIC-pos	HMDB13127	Beta hydroxy acids and derivatives	0.15503	0.0088	0.2749
C14:0 SM	C8-pos	HMDB12097	Phosphosphingolipids	-0.1546	0.0089	0.2749
Cystathionine	HILIC-neg	HMDB00099	Amino acids, peptides, and analogues	0.15438	0.0090	0.2749
Hydroxyphenylacetate	HILIC-neg	HMDB00020	1-hydroxy-2-unsubstituted benzenoids	0.15257	0.0099	0.2749
Putrescine	HILIC-pos	HMDB01414	Amines	0.15058	0.0109	0.2749
1-methylnicotinamide	HILIC-pos	HMDB00699	Pyridinecarboxylic acids and derivatives	-0.15012	0.0112	0.2749
Asparagine	HILIC-pos	HMDB00168	Amino acids, peptides, and analogues	-0.14947	0.0115	0.2749
Leucine	HILIC-pos	HMDB00687	Amino acids, peptides, and analogues	-0.14326	0.0155	0.3452
5-aminolevulinic acid	HILIC-pos	HMDB01149	Amino acids, peptides, and analogues	0.14092	0.0173	0.3609
Inosine	HILIC-neg	HMDB00195	------	0.13682	0.0209	0.3856
Histidine	HILIC-pos	HMDB00177	Amino acids, peptides, and analogues	-0.13248	0.0253	0.3856
C34:3 PE plasmalogen	C8-pos	HMDB11343	Glycerophosphoethanolamines	-0.13177	0.0261	0.3856
Symmetric dimethylarginine (SDMA)	HILIC-pos	HMDB03334	Amino acids, peptides, and analogues	0.13115	0.0268	0.3856
C24:1 ceramide (d18:1)	C8-pos	HMDB04953	Ceramides	0.1306	0.0275	0.3856
C36:4 PE	C8-pos	HMDB08937	Glycerophosphoethanolamines	0.13007	0.0281	0.3856
Urate	HILIC-neg	HMDB00289	Purines and purine derivatives	0.12993	0.0283	0.3856
C18:2 CE	C8-pos	HMDB00610	Steroid esters	-0.1296	0.0287	0.3856
Trimethylamine-N-oxide	HILIC-pos	HMDB00925	Aminoxides	0.12779	0.0310	0.3856
2-hydroxyglutarate	HILIC-neg	HMDB00694	Short-chain hydroxy acids and derivatives	0.12701	0.0321	0.3856
C24:0 SM	C8-pos	HMDB11697	Phosphosphingolipids	-0.12688	0.0323	0.3856
Fumarate	HILIC-neg	HMDB00134	Dicarboxylic acids and derivatives	0.12683	0.0323	0.3856
C22:0 SM	C8-pos	HMDB12103	Phosphosphingolipids	-0.12543	0.0343	0.3906
C20:5 LPC	C8-pos	HMDB10397	Glycerophosphocholines	-0.12489	0.0351	0.3906
Salicylurate	HILIC-neg	HMDB00840	Benzoic acids and derivatives	0.12302	0.0379	0.4088
Homogentisate	HILIC-neg	HMDB00130	Phenylacetic acids	0.12222	0.0392	0.4093
Glycodeoxycholate	HILIC-neg	HMDB00631	Bile acids, alcohols and derivatives	-0.12091	0.0414	0.4187
Malate	HILIC-neg	HMDB00156	Beta hydroxy acids and derivatives	0.11935	0.0441	0.4224
5-hydroxytryptophan	HILIC-pos	HMDB00472	Tryptamines and derivatives	-0.11925	0.0443	0.4224
C54:10 TAG	C8-pos	------	Triradylcglycerols	-0.1183	0.0460	0.4268
C44:13 PE plasmalogen	C8-pos	------	Glycerophosphoethanolamines	-0.11676	0.0489	0.4404
C18:2 LPC	C8-pos	HMDB10386	Glycerophosphocholines	-0.11582	0.0508	0.4404
C6 carnitine	HILIC-pos	HMDB00705	Fatty acid esters	0.1155	0.0514	0.4404
Uracil	HILIC-neg	HMDB00300	Pyrimidines and pyrimidine derivatives	0.11449	0.0535	0.4432
C9 carnitine	HILIC-pos	HMDB13288	Beta hydroxy acids and derivatives	-0.11394	0.0547	0.4432
Oxalate	HILIC-neg	HMDB02329	Dicarboxylic acids and derivatives	0.11346	0.0557	0.4432
C36:1 PS plasmalogen	C8-pos	------	------	0.11138	0.0604	0.4691
Kynurenic acid	HILIC-pos	HMDB00715	Quinoline carboxylic acids	0.10953	0.0648	0.4921
C36:4 PC-A	C8-pos	HMDB07983	Glycerophosphocholines	-0.10665	0.0722	0.5101
Aconitate	HILIC-neg	HMDB00072	Tricarboxylic acids and derivatives	0.10572	0.0748	0.5101
Indoxylsulfate	HILIC-neg	HMDB00682	Arylsulfates	0.10511	0.0765	0.5101
C38:2 PE	C8-pos	HMDB08942	Glycerophosphoethanolamines	-0.10482	0.0773	0.5101
C34:0 PE	C8-pos	HMDB08925	Glycerophosphoethanolamines	-0.10461	0.0779	0.5101
Quinolinate	HILIC-neg	HMDB00232	Pyridinecarboxylic acids and derivatives	0.10423	0.0790	0.5101
C36:2 PS plasmalogen	C8-pos	------	------	0.10367	0.0806	0.5101
C14:0 LPC	C8-pos	HMDB10379	Glycerophosphocholines	-0.10343	0.0813	0.5101
Fructose	HILIC-neg	HMDB00122	Carbohydrates and carbohydrate conjugates	0.10344	0.0813	0.5101
Lactose	HILIC-neg	HMDB00186	Carbohydrates and carbohydrate conjugates	0.10298	0.0826	0.5101
Lysine	HILIC-pos	HMDB00182	Amino acids, peptides, and analogues	-0.10253	0.0840	0.5101

Log-transformed and standardized Metabolites	Metabolite profiling method*	Human Metabolome Database ID	Human Metabolome Database Taxonomy sub class	Continuous SAVE scores		
				Partial Pearson correlation	p-value	False discovery rate
C26 carnitine	HILIC-pos	HMDB06347	Fatty acid esters	-0.10164	0.0868	0.5174
C20:0 SM	C8-pos	HMDB12102	Phosphosphingolipids	-0.0996	0.0933	0.5446
Isoleucine	HILIC-pos	HMDB00172	Amino acids, peptides, and analogues	-0.09899	0.0953	0.5446
Uridine	HILIC-neg	HMDB00296	------	-0.09873	0.0962	0.5446
C34:2 PC	C8-pos	HMDB07973	Glycerophosphocholines	-0.09781	0.0994	0.5480
C22:6 LPC	C8-pos	HMDB10404	Glycerophosphocholines	-0.0976	0.1001	0.5480
Methylmalonate	HILIC-neg	HMDB00202	Dicarboxylic acids and derivatives	0.09657	0.1037	0.5588
Methionine sulfoxide	HILIC-pos	HMDB02005	Amino acids, peptides, and analogues	-0.09552	0.1076	0.5636
N-Monomethyl-arginine (NMMA)	HILIC-pos	HMDB29416	Amino acids, peptides, and analogues	-0.09507	0.1093	0.5636
Pyroglutamic acid	HILIC-pos	HMDB00267	Amino acids, peptides, and analogues	-0.09496	0.1097	0.5636
C38:3 PE plasmalogen	C8-pos	HMDB11384	Glycerophosphoethanolamines	-0.09209	0.1209	0.5917
C36:2 PC	C8-pos	HMDB08039	Glycerophosphocholines	-0.09193	0.1215	0.5917
C22:1 SM	C8-pos	HMDB12104	Phosphosphingolipids	-0.09185	0.1218	0.5917
C24:0 ceramide (d18:1)	C8-pos	HMDB04956	Ceramides	-0.09176	0.1222	0.5917
C38:4 PE	C8-pos	HMDB09003	Glycerophosphoethanolamines	0.0903	0.1283	0.6012
Glycocholate	HILIC-neg	HMDB00138	Bile acids, alcohols and derivatives	-0.09026	0.1285	0.6012
C38:7 PE plasmalogen	C8-pos	HMDB11420	Glycerophosphoethanolamines	-0.09	0.1296	0.6012
C40:7 PE plasmalogen	C8-pos	HMDB11394	Glycerophosphoethanolamines	-0.08898	0.1340	0.6130
Beta-hydroxybutyrate	HILIC-neg	HMDB00011	Beta hydroxy acids and derivatives	0.08789	0.1389	0.6184
Glutamine	HILIC-pos	HMDB00641	Amino acids, peptides, and analogues	-0.0874	0.1411	0.6184
Sarcosine	HILIC-pos	HMDB00271	Amino acids, peptides, and analogues	-0.08683	0.1437	0.6184
Thyroxine	HILIC-pos	HMDB00248	Amino acids, peptides, and analogues	-0.08651	0.1452	0.6184
C34:1 PC plasmalogen-A	C8-pos	HMDB11208	Glycerophosphocholines	0.08626	0.1463	0.6184
Mevalonic acid	HILIC-pos	HMDB00227	Fatty acids and conjugates	-0.08611	0.147	0.6184
C18:0 LPC	C8-pos	HMDB10384	Glycerophosphocholines	-0.08567	0.1491	0.6184
C4 carnitine	HILIC-pos	HMDB02013	Fatty acid esters	0.0855	0.1500	0.6184
C36:4 PE plasmalogen	C8-pos	HMDB11442	Glycerophosphoethanolamines	-0.08436	0.1555	0.6263
C16:0 ceramide (d18:1)	C8-pos	HMDB04949	Ceramides	0.08433	0.1556	0.6263
Pyruvate	HILIC-neg	HMDB00243	Alpha-keto acids and derivatives	0.08354	0.1596	0.6345
C34:0 PI	C8-pos	HMDB09805	Glycerophosphoinositols	-0.0828	0.1633	0.6417
Alpha-glycerophosphate	HILIC-neg	HMDB00126	Glycerophosphates	-0.0819	0.1679	0.6522
Adenosine diphosphate (ADP)	HILIC-neg	HMDB01341	Purine ribonucleotides	0.08085	0.1735	0.6649
Carnitine	HILIC-pos	HMDB00062	Quaternary ammonium salts	-0.08053	0.1752	0.6649
Gentisate	HILIC-neg	HMDB00152	Benzoic acids and derivatives	0.08015	0.1772	0.6651
Phosphocreatine	HILIC-neg	HMDB01511	Amino acids, peptides, and analogues	-0.07826	0.1877	0.6933
C20:5 CE	C8-pos	HMDB06731	Steroid esters	-0.07805	0.1889	0.6933
Cotinine	HILIC-pos	HMDB01046	Pyrrolidinylpyridines	0.07736	0.1928	0.7001
C34:2 PE	C8-pos	HMDB08928	Glycerophosphoethanolamines	0.07618	0.1998	0.7143
hexose monophosphate	HILIC-neg	HMDB00124	Carbohydrates and carbohydrate conjugates	0.07577	0.2022	0.7143
phenylalanine	HILIC-pos	HMDB00159	Amino acids, peptides, and analogues	-0.0756	0.2032	0.7143
C32:2 PC	C8-pos	HMDB07874	Glycerophosphocholines	-0.07488	0.2075	0.722
C38:4 DAG	C8-pos	HMDB07170	Diradylglycerols	0.07357	0.2157	0.7222
sucrose	HILIC-neg	HMDB00258	Carbohydrates and carbohydrate conjugates	0.07344	0.2164	0.7222
C32:1 PI	C8-pos	HMDB09779	Glycerophosphoinositols	0.07332	0.2172	0.7222
C18:1 carnitine	HILIC-pos	HMDB05065	Fatty acid esters	0.07327	0.2175	0.7222
C34:2 PI	C8-pos	HMDB09784	Glycerophosphoinositols	-0.07279	0.2205	0.7222
C36:0 PE	C8-pos	HMDB08991	Glycerophosphoethanolamines	-0.07249	0.2225	0.7222
C56:4 TAG	C8-pos	HMDB05398	Triradylcglycerols	0.07245	0.2227	0.7222
C36:4 PC plasmalogen	C8-pos	HMDB11310	Glycerophosphocholines	0.07077	0.2337	0.7266
xanthosine	HILIC-pos	HMDB00299	Pyrimidine nucleotide sugars	-0.07068	0.2343	0.7266
C38:5 PE	C8-pos	HMDB09069	Glycerophosphoethanolamines	0.07015	0.2378	0.7266
arginine	HILIC-pos	HMDB00517	Amino acids, peptides, and analogues	-0.06993	0.2393	0.7266
C20:3 CE	C8-pos	HMDB06736	Steroid esters	-0.06966	0.2411	0.7266
C34:4 PC	C8-pos	HMDB07883	Glycerophosphocholines	-0.06963	0.2413	0.7266
betaine	HILIC-pos	HMDB00043	Amino acids, peptides, and analogues	-0.0693	0.2435	0.7266
acetylcholine	HILIC-pos	HMDB00895	Quaternary ammonium salts	0.06929	0.2436	0.7266
niacinamide	HILIC-pos	HMDB01406	Pyridinecarboxylic acids and derivatives	-0.06928	0.2437	0.7266
C36:3 PE plasmalogen	C8-pos	HMDB11441	Glycerophosphoethanolamines	-0.0688	0.2470	0.7300
beta-alanine	HILIC-pos	HMDB00056	Amino acids, peptides, and analogues	-0.06703	0.2594	0.7599
Adenosine monophosphate (AMP)	HILIC-neg	HMDB00045	Purine ribonucleotides	0.0663	0.2646	0.7678
C38:7 PC plasmalogen	C8-pos	HMDB11229	Glycerophosphocholines	-0.06579	0.2683	0.7678

Log-transformed and standardized Metabolites	Metabolite profiling method*	Human Metabolome Database ID	Human Metabolome Database Taxonomy sub class	Continuous SAVE scores		
				Partial Pearson correlation	p-value	False discovery rate
4-hydroxymandelate	HILIC-neg	HMDB00822	1-hydroxy-2-unsubstituted benzenoids	0.06564	0.2694	0.7678
C40:6 PE	C8-pos	HMDB09012	Glycerophosphoethanolamines	0.06525	0.2723	0.7678
cytosine	HILIC-pos	HMDB00630	Pyrimidines and pyrimidine derivatives	0.06507	0.2736	0.7678
C34:2 PC plasmalogen	C8-pos	HMDB11210	Glycerophosphocholines	0.06373	0.2836	0.7850
Guanosine monophosphate (GMP)	HILIC-neg	HMDB01397	Eicosanoids	0.06358	0.2848	0.7850
C36:1 PE	C8-pos	HMDB08993	Glycerophosphoethanolamines	0.06332	0.2867	0.7850
C34:3 PC plasmalogen	C8-pos	HMDB11211	Glycerophosphocholines	-0.06241	0.2937	0.7967
C34:2 PE plasmalogen	C8-pos	HMDB08952	Glycerophosphoethanolamines	-0.06193	0.2975	0.7967
C42:11 PE plasmalogen	C8-pos	------	Glycerophosphoethanolamines	-0.06184	0.2982	0.7967
C2 carnitine	HILIC-pos	HMDB00201	Fatty acid esters	0.06131	0.3024	0.8015
C36:5 PC plasmalogen-A	C8-pos	HMDB11221	Glycerophosphocholines	-0.0607	0.3072	0.8079
C38:5 DAG	C8-pos	HMDB07199	Diradylglycerols	0.06012	0.3119	0.8138
pantothenate	HILIC-neg	HMDB00210	Alcohols and polyols	-0.05962	0.3159	0.8173
C38:6 PC	C8-pos	HMDB07991	Glycerophosphocholines	-0.05935	0.3181	0.8173
C56:1 TAG	C8-pos	HMDB05396	Triradylcglycerols	-0.05848	0.3252	0.8199
C22:5 CE	C8-pos	HMDB10375	Steroid esters	-0.05792	0.3299	0.8199
C30:0 PC	C8-pos	HMDB07869	Glycerophosphocholines	-0.05789	0.3301	0.8199
C16:0 LPC	C8-pos	HMDB10382	Glycerophosphocholines	-0.05785	0.3305	0.8199
C18 carnitine	HILIC-pos	HMDB00848	Fatty acid esters	-0.05746	0.3337	0.8199
C22:0 ceramide (d18:1)	C8-pos	HMDB04952	Ceramides	-0.05735	0.3347	0.8199
C20:3 LPC	C8-pos	HMDB10393	Glycerophosphocholines	-0.05619	0.3446	0.8199
C52:0 TAG	C8-pos	HMDB05365	Triradylcglycerols	-0.05615	0.3449	0.8199
C52:2 TAG	C8-pos	HMDB05369	Triradylcglycerols	0.05612	0.3452	0.8199
C54:1 TAG	C8-pos	HMDB05395	Triradylcglycerols	-0.05607	0.3456	0.8199
C36:2 DAG	C8-pos	HMDB07218	Diradylglycerols	0.05601	0.3461	0.8199
C50:2 TAG	C8-pos	HMDB05377	Triradylcglycerols	0.0554	0.3514	0.8200
C40:9 PC	C8-pos	HMDB08731	Glycerophosphocholines	-0.05516	0.3535	0.8200
Malondialdehyde (MDA)	HILIC-neg	HMDB06112	Carbonyl compounds	-0.05493	0.3555	0.8200
histamine	HILIC-pos	HMDB00870	Amines	0.0546	0.3584	0.8200
C3-DC-CH3 carnitine	HILIC-pos	HMDB13133	Beta hydroxy acids and derivatives	0.05448	0.3595	0.8200
C18:2 LPE	C8-pos	HMDB11507	Glycerophosphoethanolamines	-0.05411	0.3628	0.8200
C36:2 PE plasmalogen	C8-pos	HMDB09082	Glycerophosphoethanolamines	-0.05404	0.3634	0.8200
C22:6 CE	C8-pos	HMDB06733	Steroid esters	-0.05318	0.3710	0.8236
hypoxanthine	HILIC-neg	HMDB00157	Purines and purine derivatives	0.05311	0.3717	0.8236
C36:5 PC plasmalogen-B	C8-pos	HMDB11220	Glycerophosphocholines	0.05304	0.3724	0.8236
citrate	HILIC-neg	HMDB00094	Tricarboxylic acids and derivatives	0.05265	0.3758	0.8259
C46:0 TAG	C8-pos	HMDB10411	Triradylcglycerols	-0.05212	0.3807	0.8267
C20:4 LPC	C8-pos	HMDB10395	Glycerophosphocholines	-0.05207	0.3812	0.8267
C56:6 TAG	C8-pos	HMDB05456	Triradylcglycerols	0.05142	0.3871	0.8342
C38:6 PE	C8-pos	HMDB09102	Glycerophosphoethanolamines	0.05076	0.3933	0.8390
C54:6 TAG	C8-pos	HMDB05391	Triradylcglycerols	0.05064	0.3944	0.8390
C34:1 PC	C8-pos	HMDB07972	Glycerophosphocholines	0.05032	0.3974	0.8400
valine	HILIC-pos	HMDB00883	Amino acids, peptides, and analogues	-0.04926	0.4074	0.8559
Guanosine diphosphate (GDP)	HILIC-neg	HMDB01201	Purine ribonucleotides	0.04866	0.4131	0.8617
inositol	HILIC-neg	HMDB00211	Alcohols and polyols	0.04843	0.4154	0.8617
C16 carnitine	HILIC-pos	HMDB00222	Fatty acid esters	0.04783	0.4212	0.8653
C32:1 PC	C8-pos	HMDB07873	Glycerophosphocholines	0.04764	0.4231	0.8653
C8 carnitine	HILIC-pos	HMDB00791	Fatty acid esters	0.04722	0.4272	0.8653
2-phosphoglycerate	HILIC-neg	HMDB00807	Carbohydrates and carbohydrate conjugates	-0.04719	0.4275	0.8653
C34:2 DAG	C8-pos	HMDB07103	Lineolic acids and derivatives	0.04651	0.4341	0.8734
C30:0 DAG	C8-pos	HMDB07011	Diradylglycerols	-0.04482	0.4510	0.9020
C16:0 CE	C8-pos	HMDB00885	Steroid esters	-0.04452	0.4540	0.9027
alpha-hydroxybutyrate	HILIC-neg	HMDB00008	Alpha hydroxy acids and derivatives	0.04343	0.4652	0.9049
succinate	HILIC-neg	HMDB00254	Dicarboxylic acids and derivatives	0.04319	0.4676	0.9049
C10 carnitine	HILIC-pos	HMDB00651	Fatty acid esters	0.04302	0.4695	0.9049
sorbitol	HILIC-neg	HMDB00247	Carbohydrates and carbohydrate conjugates	0.04231	0.4768	0.9049
C52:3 TAG	C8-pos	HMDB05384	Triradylcglycerols	0.04212	0.4788	0.9049
threonine	HILIC-pos	HMDB00167	Amino acids, peptides, and analogues	0.04184	0.4817	0.9049
C54:4 TAG	C8-pos	HMDB05370	Triradylcglycerols	0.04148	0.4855	0.9049
C40:10 PC	C8-pos	HMDB08511	Glycerophosphocholines	-0.04123	0.4882	0.9049
taurine	HILIC-pos	HMDB00251	Organosulfonic acids and derivatives	0.04119	0.4886	0.9049

Log-transformed and standardized Metabolites	Metabolite profiling method*	Human Metabolome Database ID	Human Metabolome Database Taxonomy sub class	Continuous SAVE scores		
				Partial Pearson correlation	p-value	False discovery rate
choline	HILIC-pos	HMDB00097	Quaternary ammonium salts	-0.04083	0.4924	0.9049
Uridine diphosphate (UDP)-glucose	HILIC-neg	HMDB00286	Pyrimidine nucleotide sugars	0.04035	0.4975	0.9049
C56:3 TAG	C8-pos	HMDB05410	Triradylcglycerols	0.04015	0.4996	0.9049
proline	HILIC-pos	HMDB00162	Amino acids, peptides, and analogues	0.04007	0.5005	0.9049
2-aminoadipate	HILIC-neg	HMDB00510	Amino acids, peptides, and analogues	0.03963	0.5052	0.9049
3-hydroxyanthranilic acid	HILIC-pos	HMDB01476	Benzoic acids and derivatives	-0.03955	0.5061	0.9049
C36:2 PE	C8-pos	HMDB08994	Glycerophosphoethanolamines	0.03952	0.5064	0.9049
C16-OH carnitine	HILIC-pos	HMDB13337	Beta hydroxy acids and derivatives	-0.03906	0.5113	0.9049
C36:0 PC	C8-pos	HMDB08036	Glycerophosphocholines	-0.03904	0.5115	0.9049
C5-DC carnitine	HILIC-pos	HMDB13130	Beta hydroxy acids and derivatives	0.03896	0.5124	0.9049
C24:1 SM	C8-pos	HMDB12107	Phosphosphingolipids	0.0388	0.5141	0.9049
allantoin	HILIC-pos	HMDB00462	Imidazoles	0.03864	0.5159	0.9049
C54:3 TAG	C8-pos	HMDB05405	Triradylcglycerols	0.03821	0.5205	0.9049
C48:5 TAG	C8-pos	------	Triradylcglycerols	-0.03803	0.5226	0.9049
C56:5 TAG	C8-pos	HMDB05406	Triradylcglycerols	0.038	0.5228	0.9049
C34:0 DAG	C8-pos	HMDB07100	Diradylglycerols	-0.038	0.5229	0.9049
phosphoethanolamine	HILIC-pos	HMDB00224	Phosphate esters	0.03739	0.5296	0.9118
C34:0 PS	C8-pos	HMDB12356	Glycerophosphoserines	-0.03705	0.5333	0.9135
citrulline	HILIC-pos	HMDB00904	Amino acids, peptides, and analogues	0.03573	0.5480	0.9273
C22:6 LPE	C8-pos	HMDB11526	Glycerophosphoethanolamines	-0.03533	0.5525	0.9273
C20:4 LPE	C8-pos	HMDB11517	Glycerophosphoethanolamines	0.03524	0.5535	0.9273
hydroxyproline	HILIC-pos	HMDB00725	Amino acids, peptides, and analogues	0.03512	0.5549	0.9273
C44:0 TAG	C8-pos	HMDB42063	Triradylcglycerols	-0.03508	0.5553	0.9273
C42:0 TAG	C8-pos	------	Triradylcglycerols	-0.03473	0.5592	0.9293
alpha-glycerophosphocholine	HILIC-pos	HMDB00086	Glycerophosphocholines	-0.03444	0.5625	0.9294
C38:6 PE plasmalogen	C8-pos	HMDB11387	Glycerophosphoethanolamines	-0.03415	0.5659	0.9294
C38:2 PC	C8-pos	HMDB08270	Glycerophosphocholines	-0.03372	0.5707	0.9294
C34:3 DAG	C8-pos	HMDB07132	Lineolic acids and derivatives	0.03342	0.5743	0.9294
C36:3 PE	C8-pos	HMDB09060	Glycerophosphoethanolamines	0.0333	0.5756	0.9294
chenodeoxycholate/deoxycholate	HILIC-neg	HMDB00626	Bile acids, alcohols and derivatives	0.03285	0.5808	0.9294
hippurate	HILIC-neg	HMDB00714	Benzoic acids and derivatives	0.0327	0.5825	0.9294
C16:0 LPE	C8-pos	HMDB11503	Glycerophosphoethanolamines	0.03255	0.5842	0.9294
C44:1 TAG	C8-pos	------	Triradylcglycerols	-0.0322	0.5882	0.9294
C56:2 TAG	C8-pos	HMDB05404	Triradylcglycerols	-0.03215	0.5888	0.9294
C36:3 DAG	C8-pos	HMDB07219	Lineolic acids and derivatives	0.03187	0.5921	0.9294
C50:0 TAG	C8-pos	HMDB05357	Triradylcglycerols	-0.03178	0.5932	0.9294
C32:0 PC	C8-pos	HMDB07871	Glycerophosphocholines	0.03142	0.5974	0.9294
cAMP	HILIC-neg	HMDB00058	Cyclic purine nucleotides	0.03134	0.5983	0.9294
C18:3 CE	C8-pos	HMDB10370	Steroid esters	-0.03035	0.6098	0.9423
C50:1 TAG	C8-pos	HMDB05360	Triradylcglycerols	0.02986	0.6156	0.9423
C34:0 PC	C8-pos	HMDB07970	Glycerophosphocholines	-0.02931	0.6222	0.9423
C46:4 TAG	C8-pos	------	Triradylcglycerols	-0.02924	0.623	0.9423
C18:2 carnitine	HILIC-pos	HMDB06469	Fatty acid esters	0.02885	0.6277	0.9423
Asymmetric dimethylarginine (ADMA)	HILIC-pos	HMDB01539	Amino acids, peptides, and analogues	-0.02843	0.6326	0.9423
alanine	HILIC-pos	HMDB00161	Amino acids, peptides, and analogues	0.0284	0.6330	0.9423
ornithine	HILIC-pos	HMDB00214	Amino acids, peptides, and analogues	-0.02814	0.6361	0.9423
sphingosine	C8-pos	HMDB00252	Amines	0.0281	0.6367	0.9423
C50:3 TAG	C8-pos	HMDB05433	Triradylcglycerols	0.02793	0.6387	0.9423
C18:1 LPC	C8-pos	HMDB02815	Glycerophosphocholines	-0.02744	0.6446	0.9423
C38:4 PI	C8-pos	HMDB09815	Glycerophosphoinositols	-0.02734	0.6458	0.9423
C16:1 CE	C8-pos	HMDB00658	Steroid esters	0.02679	0.6525	0.9423
Gamma-Aminobutyric acid (GABA)	HILIC-pos	HMDB00112	Amino acids, peptides, and analogues	-0.02651	0.6559	0.9423
C44:2 TAG	C8-pos	------	Triradylcglycerols	-0.02633	0.6580	0.9423
C48:0 TAG	C8-pos	HMDB05356	Triradylcglycerols	-0.02627	0.6588	0.9423
C18:0 LPE	C8-pos	HMDB11130	Glycerophosphoethanolamines	0.02584	0.6640	0.9423
Inosinic acid (IMP)	HILIC-neg	HMDB00175	Purine ribonucleotides	0.02572	0.6655	0.9423
C18:1 SM	C8-pos	HMDB12101	Phosphosphingolipids	0.02559	0.6671	0.9423
C46:2 TAG	C8-pos	HMDB10419	Triradylcglycerols	-0.02539	0.6696	0.9423
C18:2 SM	C8-pos	------	------	0.02494	0.6750	0.9423

Log-transformed and standardized Metabolites	Metabolite profiling method*	Human Metabolome Database ID	Human Metabolome Database Taxonomy sub class	Continuous SAVE scores		
				Partial Pearson correlation	p-value	False discovery rate
xanthine	HILIC-neg	HMDB00292	Purines and purine derivatives	-0.02486	0.6760	0.9423
C46:3 TAG	C8-pos	------	Triradylcglycerols	-0.02467	0.6783	0.9423
aminoisobutyric acid	HILIC-pos	HMDB01906	Amino acids, peptides, and analogues	0.02437	0.6820	0.9423
C14:1 carnitine	HILIC-pos	HMDB02014	Fatty acid esters	0.02386	0.6883	0.9423
C22:1 MAG	C8-pos	HMDB11582	Monoradylglycerols	0.0238	0.6891	0.9423
C52:5 TAG	C8-pos	HMDB05380	Triradylcglycerols	0.02357	0.6919	0.9423
C36:3 PC	C8-pos	HMDB08105	Glycerophosphocholines	-0.02337	0.6944	0.9423
C36:0 DAG	C8-pos	HMDB07158	Diradylglycerols	0.02315	0.6971	0.9423
Creatine	HILIC-pos	HMDB00064	Amino acids, peptides, and analogues	0.02304	0.6985	0.9423
C58:7 TAG	C8-pos	HMDB05471	Triradylcglycerols	0.02216	0.7095	0.9423
C5 carnitine	HILIC-pos	HMDB00688	Fatty acid esters	-0.02202	0.7112	0.9423
C14:1 MAG	C8-pos	HMDB11562	Monoradylglycerols	-0.02182	0.7137	0.9423
C18:0 MAG	C8-pos	HMDB11131	Monoradylglycerols	-0.02173	0.7149	0.9423
C56:8 TAG	C8-pos	HMDB05392	Triradylcglycerols	-0.02114	0.7223	0.9423
C18:1-OH carnitine	HILIC-pos	HMDB13339	Beta hydroxy acids and derivatives	0.02104	0.7236	0.9423
Serine	HILIC-pos	HMDB00187	Amino acids, peptides, and analogues	-0.0209	0.7253	0.9423
C7 carnitine	HILIC-pos	HMDB13238	Beta hydroxy acids and derivatives	0.02088	0.7256	0.9423
C12 carnitine	HILIC-pos	HMDB02250	Fatty acid esters	-0.02084	0.7261	0.9423
C36:2 PC plasmalogen	C8-pos	HMDB11243	Glycerophosphocholines	0.02063	0.7287	0.9423
Lactate	HILIC-neg	HMDB00190	Alpha hydroxy acids and derivatives	0.02005	0.7361	0.9423
C18:0 CE	C8-pos	HMDB10368	Steroid esters	-0.01977	0.7396	0.9423
C52:7 TAG	C8-pos	HMDB10517	Triradylcglycerols	-0.01978	0.7396	0.9423
C36:1 PC plasmalogen	C8-pos	HMDB11241	Glycerophosphocholines	0.01909	0.7483	0.9423
C46:1 TAG	C8-pos	HMDB10412	Triradylcglycerols	-0.01904	0.7489	0.9423
C38:5 PE plasmalogen	C8-pos	HMDB11386	Glycerophosphoethanolamines	0.0189	0.7507	0.9423
C16:1 SM	C8-pos	------	------	0.01881	0.7519	0.9423
C16:1 MAG	C8-pos	HMDB11565	Monoradylglycerols	0.01878	0.7522	0.9423
C36:5 PE plasmalogen	C8-pos	HMDB11410	Glycerophosphoethanolamines	-0.01874	0.7528	0.9423
Glutamate	HILIC-pos	HMDB00148	Amino acids, peptides, and analogues	0.01867	0.7537	0.9423
C38:4 PC plasmalogen	C8-pos	HMDB11252	Glycerophosphocholines	0.01861	0.7544	0.9423
C38:3 PC	C8-pos	HMDB08047	Glycerophosphocholines	-0.01839	0.7572	0.9423
C58:11 TAG	C8-pos	HMDB10531	Triradylcglycerols	-0.01816	0.7601	0.9423
C56:9 TAG	C8-pos	HMDB05448	Triradylcglycerols	-0.01813	0.7605	0.9423
Uridine 5'-diphosphate (UDP)	HILIC-neg	HMDB00295	Pyrimidine ribonucleotides	0.01804	0.7617	0.9423
C40:6 PC	C8-pos	HMDB08057	Glycerophosphocholines	-0.0164	0.7829	0.9546
C48:1 TAG	C8-pos	HMDB05359	Triradylcglycerols	-0.01625	0.7847	0.9546
C34:1 DAG	C8-pos	HMDB07102	Diradylglycerols	0.01603	0.7875	0.9546
C52:4 TAG	C8-pos	HMDB05363	Triradylcglycerols	0.01586	0.7898	0.9546
C36:3 PC plasmalogen	C8-pos	HMDB11244	Glycerophosphocholines	-0.01563	0.7928	0.9546
C54:8 TAG	C8-pos	HMDB10518	Triradylcglycerols	-0.0152	0.7984	0.9546
Adenosine	HILIC-pos	HMDB00050	------	0.01511	0.7995	0.9546
C58:9 TAG	C8-pos	HMDB05463	Triradylcglycerols	-0.01443	0.8084	0.9546
C38:6 PC plasmalogen	C8-pos	HMDB11319	Glycerophosphocholines	0.01437	0.8092	0.9546
3-methyladipate/pimelate	HILIC-neg	HMDB00555	Fatty acids and conjugates	-0.01384	0.8160	0.9546
C58:6 TAG	C8-pos	HMDB05458	Triradylcglycerols	0.01384	0.8160	0.9546
Acetylglycine	HILIC-pos	HMDB00532	Amino acids, peptides, and analogues	-0.01368	0.8181	0.9546
C16:1 LPC	C8-pos	HMDB10383	Glycerophosphocholines	0.01341	0.8217	0.9546
C20:4 CE	C8-pos	HMDB06726	Steroid esters	-0.01331	0.8230	0.9546
Serotonin	HILIC-pos	HMDB00259	Tryptamines and derivatives	0.01276	0.8301	0.9546
C48:4 TAG	C8-pos	------	Triradylcglycerols	-0.01272	0.8307	0.9546
1-methylhistamine	HILIC-pos	HMDB00898	Amines	-0.01246	0.8342	0.9546
C32:2 DAG	C8-pos	HMDB07128	Diradylglycerols	0.01245	0.8342	0.9546
Benzoate	HILIC-neg	HMDB01870	Benzoic acids and derivatives	0.01238	0.8351	0.9546
C14 carnitine	HILIC-pos	HMDB05066	Fatty acid esters	-0.01187	0.8419	0.9546
C36:1 DAG	C8-pos	HMDB07216	Diradylglycerols	-0.0116	0.8454	0.9546
C54:7 TAG	C8-pos	HMDB05447	Triradylcglycerols	0.0116	0.8455	0.9546
C50:6 TAG	C8-pos	HMDB10497	Triradylcglycerols	-0.01156	0.8459	0.9546
Dimethylglycine	HILIC-pos	HMDB00092	Amino acids, peptides, and analogues	0.01154	0.8462	0.9546
C48:3 TAG	C8-pos	HMDB05432	Triradylcglycerols	-0.01145	0.8474	0.9546
C34:3 PC	C8-pos	HMDB08006	Glycerophosphocholines	-0.0114	0.8481	0.9546
C16:0 SM	C8-pos	HMDB10169	Phosphosphingolipids	0.01123	0.8503	0.9546
C36:1 PC	C8-pos	HMDB08038	Glycerophosphocholines	0.01111	0.8518	0.9546

Log-transformed and standardized Metabolites	Metabolite profiling method*	Human Metabolome Database ID	Human Metabolome Database Taxonomy sub class	Continuous SAVE scores		
				Partial Pearson correlation	p-value	False discovery rate
C18:0 SM	C8-pos	HMDB01348	Phosphosphingolipids	0.01091	0.8545	0.9546
C54:5 TAG	C8-pos	HMDB05385	Triradylcglycerols	0.01062	0.8583	0.9556
C58:10 TAG	C8-pos	HMDB05476	Triradylcglycerols	-0.01008	0.8655	0.9604
C10:2 carnitine	HILIC-pos	HMDB13325	------	-0.00986	0.8684	0.9604
C18:1 CE	C8-pos	HMDB00918	Steroid esters	0.00958	0.8721	0.9606
C50:5 TAG	C8-pos	HMDB10471	Triradylcglycerols	-0.00913	0.8780	0.9606
C48:2 TAG	C8-pos	HMDB05376	Triradylcglycerols	-0.00908	0.8787	0.9606
C14:2 carnitine	HILIC-pos	HMDB13331	------	0.00898	0.8801	0.9606
Alpha-ketoglutarate	HILIC-neg	HMDB00208	Gamma-keto acids and derivatives	0.0084	0.8877	0.9658
C5:1 carnitine	HILIC-pos	HMDB02366	Fatty acid esters	-0.0081	0.8917	0.9669
Kynurenine	HILIC-neg	HMDB00684	Carbonyl compounds	-0.00789	0.8945	0.9669
C36:4 PC-B	C8-pos	HMDB08138	Glycerophosphocholines	-0.00594	0.9205	0.9785
C56:10 TAG	C8-pos	HMDB10513	Triradylcglycerols	-0.00581	0.9222	0.9785
Xanthurenate	HILIC-neg	HMDB00881	Quinoline carboxylic acids	0.00576	0.9228	0.9785
C56:7 TAG	C8-pos	HMDB05462	Triradylcglycerols	-0.00569	0.9239	0.9785
C32:2 PI	C8-pos	HMDB09798	Glycerophosphoinositols	0.00543	0.9273	0.9785
C36:4 DAG	C8-pos	HMDB07248	Lineolic acids and derivatives	0.00494	0.9338	0.9785
C18:1 LPE	C8-pos	HMDB11506	Glycerophosphoethanolamines	-0.00465	0.9377	0.9785
C40:7 PC plasmalogen	C8-pos	HMDB11294	Glycerophosphocholines	0.00465	0.9377	0.9785
C54:2 TAG	C8-pos	HMDB05403	Triradylcglycerols	0.00433	0.9419	0.9785
C52:6 TAG	C8-pos	HMDB05436	Triradylcglycerols	0.004	0.9464	0.9785
C3 carnitine	HILIC-pos	HMDB00824	Fatty acid esters	0.00388	0.9480	0.9785
C38:4 PC	C8-pos	HMDB08048	Glycerophosphocholines	0.0037	0.9505	0.9785
4-pyridoxate	HILIC-neg	HMDB00017	Pyridinecarboxylic acids and derivatives	-0.00368	0.9507	0.9785
C30:1 PC	C8-pos	HMDB07870	Glycerophosphocholines	0.00331	0.9557	0.9785
C20:4 carnitine	HILIC-pos	------	------	0.0033	0.9558	0.9785
C52:1 TAG	C8-pos	HMDB05367	Triradylcglycerols	-0.00323	0.9567	0.9785
Indole-3-propionate	HILIC-neg	HMDB02302	Indolyl carboxylic acids and derivatives	-0.00313	0.9580	0.9785
Adipate	HILIC-neg	HMDB00448	Fatty acids and conjugates	0.00305	0.9591	0.9785
C12:1 carnitine	HILIC-pos	HMDB13326	Beta hydroxy acids and derivatives	0.0027	0.9639	0.9785
Pipecolic acid	HILIC-pos	HMDB00716	Amino acids, peptides, and analogues	0.00256	0.9657	0.9785
C50:4 TAG	C8-pos	HMDB05435	Triradylcglycerols	-0.00248	0.9668	0.9785
C22:4 CE	C8-pos	HMDB06729	Steroid esters	0.00177	0.9763	0.9852
C32:0 DAG	C8-pos	HMDB07098	Diradylglycerols	0.00093	0.9875	0.9935
C58:8 TAG	C8-pos	HMDB05413	Triradylcglycerols	0.00068	0.9909	0.9939
C32:1 DAG	C8-pos	HMDB07099	Diradylglycerols	-0.00006	0.9992	0.9992

Grey shaded rows indicate metabolites with false discovery rate <0.30

*Metabolite profiling methods: Lipid profiling method (C8-pos); Positive ion mode MS detection (HILIC-pos); Negative ion mode MS detection (HILIC-neg)

Note: the following 16 metabolites were excluded because they were measured in <80% of participants: anserine, 6-phosphogluconate, adenine, anthranilic acid, butyrobetaine, C14:0 CE, C20 carnitine, C54:9 TAG, cholate, glutathione oxidized, glycine, homocysteine, orotate, phosphoenolpyruvate, propionate, and taurodeoxycholate

Table S2. Attenuation of correlation between SAVE scores and top 37 metabolites after further adjustments in addition to age and study site, organized by taxonomy classification

**Metabolites**, organized by HMDB taxonomy super and sub class	Correlation with SAVE, adjusted for age & site (N=287)	A) Adjusting for smoking status (N=287)		B) Adjusting for body mass index (N=287)		C) Adjusting for % fat and appendicular lean mass (n=275[a])		D) Adjusting for daily protein intake (n=283[a])		E) Adjusting for IL-6 and CRP (n=275[a])	
		Correlation	% attenuation	Correlation	% attenuation	Correlation	% attenuation	Correlation	% attenuation	Correlation	% attenuation
**Organic acids and derivatives:**											
Amino acids/peptides/analogues:											
Methionine	-0.19, p=.001	-0.19, p=.001	0.17%	-0.19, p=.001	-2.1%	-0.18, p=.004	5.6%	-0.17, p=.004	4.5%	-0.18, p=.004	4.1%
Tyrosine	-0.17, p=.004	-0.17, p=.004	0.18%	-0.18, p=.002	-6.9%	-0.17, p=.005	-8.2%	-0.16, p=.009	3.6%	-0.18, p=.002	-1.2%
Creatinine	0.16, p=.008	0.16, p=.007	-2.1%	0.16, p=.009	0.29%	0.15, p=.01	-6.8%	0.17, p=.004	-1.4%	0.15, p=.01	5.0%
Cystathionine	0.15, p=.009	0.15, p=.009	0.26%	0.15, p=.009	0.26%	0.16, p=.009	0.36%	0.16, p=.008	5.2%	0.14, p=.02	1.4%
Asparagine	-0.15, p=.01	-0.15, p=.01	0.30%	-0.15, p=.01	0.25%	-0.15, p=.02	5.9%	-0.15, p=.02	0.57%	-0.13, p=.03	4.5%
Leucine	-0.14, p=.02	-0.14, p=.02	0.33%	-0.16, p=.009	-8.6%	-0.14, p=.02	0.95%	-0.14, p=.02	0.71%	-0.15, p=.01	-1.6%
5-aminolevulinic acid	0.14, p=.02	0.14, p=.02	-1.9%	0.14, p=.02	0.36%	0.13, p=.04	7.7%	0.15, p=.01	0.82%	0.11, p=.06	4.9%
Histidine	-0.13, p=.03	-0.13, p=.03	0.27%	-0.13, p=.02	-1.1%	-0.13, p=.03	7.5%	-0.12, p=.048	4.4%	-0.11, p=.07	7.5%
Symmetric dimethylarginine (SDMA)	0.13, p=.03	0.13, p=.02	-1.6%	0.13, p=.02	-1.5%	0.12, p=.05	3.9%	0.15, p=.01	-1.3%	0.12, p=.04	3.1%
Beta hydroxy acids/derivatives:											
C4-OH carnitine	0.16, p=.009	0.15, p=.009	0.13%	0.16, p=.009	0.02%	0.15, p=.01	2.1%	0.17, p=.006	2.8%	0.15, p=.01	-5.1%
Malate	0.12, p=.04	0.12, p=.045	0.36%	0.12, p=.04	-2.4%	0.12, p=.048	2.3%	0.13, p=.03	3.2%	0.14, p=.02	-7.0%
Ureas:											
N-carbamoyl-beta-alanine	0.17, p=.004	0.17, p=.004	0.05%	0.17, p=.004	-0.16%	0.18, p=.002	2.7%	0.19, p=.001	2.4%	0.15, p=.01	1.5%
Tricarboxylic acids/derivatives:											
Isocitrate	0.17, p=.004	0.17, p=.004	0.06%	0.17, p=.004	0.21%	0.16, p=.007	6.1%	0.17, p=.004	7.4%	0.17, p=.004	-3.4%
Dicarboxylic acids/derivatives:											
Fumarate	0.13, p=.03	0.13, p=.03	0.33%	0.13, p=.03	-0.84%	0.15, p=.01	2.9%	0.14, p=.02	5.3%	0.14, p=.02	-8.7%
Short-chain hydroxy acids/derivatives:											
2-hydroxyglutarate	0.13, p=.03	0.13, p=.03	-0.59%	0.13, p=.03	-0.03%	0.12, p=.05	11%	0.13, p=.03	0.15%	0.13, p=.03	-0.19%
**Lipids and lipid-like molecules:**											
Phosphosphingolipids:											
C14:0 SM	-0.15, p=.009	-0.15, p=.009	0.23%	-0.16, p=.007	-3.3%	-0.16, p=.007	-7.3%	-0.15, p=.01	-2.9%	-0.15, p=.01	-1.5%
C24:0 SM	-0.13, p=.03	-0.13, p=.03	0.42%	-0.13, p=.03	-1.4%	-0.14, p=.02	-4.9%	-0.13, p=.03	0.03%	-0.10, p=.09	4.2%
C22:0 SM	-0.13, p=.03	-0.12, p=.04	0.44%	-0.13, p=.03	-2.5%	-0.15, p=.01	-8.9%	-0.14, p=.02	-2.7%	-0.11, p=.07	-0.15%
Glycerophosphoethanolamines:											
C34:3 PE plasmalogen	-0.13, p=.03	-0.13, p=.03	-0.05%	-0.13, p=.03	-0.69%	-0.14, p=.02	-4.0%	-0.12, p=.04	-2.4%	-0.09, p=.16	4.2%
C36:4 PE	0.13, p=.03	0.13, p=.03	0.22%	0.13, p=.03	-0.26%	0.12, p=.04	-10%	0.13, p=.03	-1.9%	0.13, p=.03	-2.4%
C44:13 PE plasmalogen	-0.12, p=.049	-0.12, p=.05	0.50%	-0.12, p=.05	0.39%	-0.10, p=.09	4.8%	-0.11, p=.06	-2.2%	-0.09, p=.15	8.6%
Ceramides:											
C24:1 ceramide (d18:1)	0.13, p=.03	0.13, p=.03	0.009%	0.13, p=.03	-0.74%	0.13, p=.03	0.07%	0.13, p=.02	-0.29%	0.13, p=.04	1.2%
Glycerophosphocholines:											
C20:5 LPC	-0.12, p=.04	-0.12, p=.04	0.23%	-0.13, p=.04	-0.19%	-0.12, p=.06	5.8%	-0.12, p=.04	-2.1%	-0.09, p=.14	11%
Bile acids, alcohols/derivatives:											
Glycodeoxycholate	-0.12, p=.04	-0.12, p=.04	-0.49%	-0.12, p=.04	0.50%	-0.13, p=.04	-4.9%	-0.13, p=.03	-6.9%	-0.11, p=.07	-2.3%
Triradylcglycerols:											
C54:10 TAG	-0.12, p=.046	-0.12, p=.047	0.47%	-0.12, p=.04	-3.2%	-0.15, p=.01	-5.1%	-0.12, p=.049	-0.60%	-0.11, p=.07	2.3%
Steroid esters:											
C18:2 CE	-0.13, p=.03	-0.13, p=.03	-0.14%	-0.13, p=.03	-0.91%	-0.13, p=.03	-0.33%	-0.13, p=.03	-2.2%	-0.10, p=.11	4.8%
**Organoheterocyclic compounds:**											
Indolyl carboxylic acids/derivatives:											
Tryptophan	-0.21, p=.0005	-0.20, p=.0005	0.14%	-0.21, p=.0004	-2.3%	-0.19, p=.002	5.1%	-0.20, p=.0007	-2.2%	-0.23, p=.0001	0.66%
Pyridinecarboxylic acids/derivatives:											
1-methylnicotinamide	-0.15, p=.01	-0.15, p=.01	0.29%	-0.15, p=.01	0.18%	-0.16, p=.007	-3.8%	-0.16, p=.006	-5.3%	-0.13, p=.03	4.7%
Purines and purine derivatives:											
Urate	0.13, p=.03	0.13, p=.03	0.04%	0.13, p=.03	-0.75%	0.14, p=.02	-2.8%	0.13, p=.03	3.2%	0.12, p=.047	-1.1%
Tryptamines and derivatives:											
5-hydroxytryptophan	-0.12, p=.04	-0.12, p=.04	-2.0%	-0.12, p=.04	-0.27%	-0.11, p=.07	11%	-0.12, p=.04	2.2%	-0.13, p=.04	-0.59%
**Benzenoids:**											
1-hydroxy-2-unsubstituted benzenoids:											
Hydroxyphenylacetate	0.15, p=.01	0.15, p=.01	0.29%	0.15, p=.01	0.32%	0.13, p=.03	7.1%	0.14, p=.02	-4.5%	0.16, p=.01	3.0%
Benzoic acids and derivatives:											
Salicylurate	0.12, p=.04	0.12, p=.04	0.25%	0.12, p=.04	0.10%	0.11, p=.08	11%	0.10, p=.09	4.9%	0.13, p=.03	4.3%

**Metabolites**, organized by HMDB taxonomy super and sub class	Correlation with SAVE, adjusted for age & site (N=287)	A) Adjusting for smoking status (N=287)		B) Adjusting for body mass index (N=287)		C) Adjusting for % fat and appendicular lean mass (n=275[a])		D) Adjusting for daily protein intake (n=283[a])		E) Adjusting for IL-6 and CRP (n=275[a])	
		Correlation	% attenuation	Correlation	% attenuation	Correlation	% attenuation	Correlation	% attenuation	Correlation	% attenuation
Phenylacetic acids: Homogentisate	0.12, p=.04	0.12, p=.04	-0.04%	0.12, p=.04	0.32%	0.14, p=.03	-8.6%	0.11, p=.06	9.2%	0.15, p=.01	3.2%
**Organic nitrogen compounds:** *Amines:* Putrescine	0.15, p=.01	0.16, p=.008	-4.7%	0.15, p=.01	0.03%	0.15, p=.01	-1.7%	0.13, p=.03	7.9%	0.14, p=.02	-0.10%
Aminoxides: Trimethylamine-N-oxide	0.13, p=.03	0.13, p=.03	0.28%	0.13, p=.03	0.35%	0.10, p=.11	9.5%	0.14, p=.02	-11%	0.14, p=.02	0.03%
**Nucleosides/nucleotides/analogues:** *Sub class not available:* Inosine	0.14, p=.02	0.14, p=.02	0.24%	0.14, p=.02	0.03%	0.14, p=.02	1.8%	0.13, p=.03	7.2%	0.13, p=.03	3.6%
**Organic oxygen compounds:** *Carbohydrates/carbohydrate conjugates:* Glucuronate	0.21, p=.0003	0.21, p=.0003	-0.08%	0.21, p=.0003	0.01%	0.20, p=.001	5.2%	0.22, p=.0002	3.9%	0.21, p=.0006	0.61%

Columns of Table S2 continued.

Log-transformed and standardized Metabolites	Age/site-adjusted correlation with SAVE (N=287)	F) Adjusting for creatinine (n=284[a])		G) Adjusting for prevalent diseases[b] (N=287)		H) Adjusting for medications[c] (N=287)		I) Adjusting for multiple variables[d] (n=257[a])	
		Correlation	% attenuation	Correlation	% attenuation	Correlation	% attenuation	Correlation	% attenuation
**Organic acids and derivatives:** *Amino acids/peptides/analogues:* Methionine	-0.19, p=.001	-0.17, p=.004	-0.61%	-0.17, p=.005	11%	-0.16, p=.006	13%	-0.13, p=.04	16%
Tyrosine	-0.17, p=.004	-0.15, p=.01	9.0%	-0.18, p=.003	-4.5%	-0.20, p=.0009	-14%	-0.16, p=.01	-5.1%
Creatinine	0.16, p=.008	0.04, p=.48	74%	0.13, p=.03	16%	0.11, p=.07	30%	0.02, p=.79	89%
Cystathionine	0.15, p=.009	0.14, p=.02	25%	0.18, p=.003	-15%	0.15, p=.01	1.8%	0.11, p=.09	32%
Asparagine	-0.15, p=.01	-0.15, p=.01	-2.5%	-0.13, p=.03	11%	-0.12, p=.046	21%	-0.12, p=.07	13%
Leucine	-0.14, p=.02	-0.13, p=.03	3.7%	-0.19, p=.002	-29%	-0.20, p=.0007	-41%	-0.19, p=.003	-34%
5-aminolevulinic acid	0.14, p=.02	0.04, p=.47	69%	0.11, p=.06	20%	0.10, p=.11	32%	-0.02, p=.81	113%
Histidine	-0.13, p=.03	-0.14, p=.02	-3.9%	-0.12, p=.05	12%	-0.14, p=.02	-2.3%	-0.12, p=.07	4.3%
Symmetric dimethylarginine (SDMA)	0.13, p=.03	0.02, p=.71	83%	0.12, p=.04	5.2%	0.11, p=.07	18%	0.02, p=.77	86%
Beta hydroxy acids/derivatives: C4-OH carnitine	0.16, p=.009	0.12, p=.04	15%	0.12, p=.04	23%	0.13, p=.04	21%	0.10, p=.14	35%
Malate	0.12, p=.04	0.10, p=.08	14%	0.12, p=.045	0.09%	0.16, p=.007	-35%	0.16, p=.01	-8.8%
Ureas: N-carbamoyl-beta-alanine	0.17, p=.004	0.14, p=.02	26%	0.14, p=.02	20%	0.13, p=.03	26%	0.13, p=.045	39%
Tricarboxylic acids/derivatives: Isocitrate	0.17, p=.004	0.13, p=.03	30%	0.16, p=.009	8.5%	0.15, p=.01	11%	0.11, p=.08	40%
Dicarboxylic acids/derivatives: Fumarate	0.13, p=.03	0.12, p=.05	12%	0.13, p=.03	-3.1%	0.14, p=.02	-11%	0.19, p=.002	-6.6%
Short-chain hydroxy acids/derivatives: 2-hydroxyglutarate	0.13, p=.03	0.06, p=.28	47%	0.11, p=.07	15%	0.12, p=.04	4.2%	0.07, p=.29	49%
**Lipids and lipid-like molecules:** *Phosphosphingolipids:* C14:0 SM	-0.15, p=.009	-0.15, p=.01	-0.32%	-0.16, p=.009	-0.73%	-0.16, p=.009	-0.62%	-0.14, p=.03	-6.2%
C24:0 SM	-0.13, p=.03	-0.14, p=.02	-7.3%	-0.12, p=.04	2.4%	-0.13, p=.03	0.86%	-0.13, p=.049	-2.5%
C22:0 SM	-0.13, p=.03	-0.14, p=.02	-6.4%	-0.12, p=.04	2.4%	-0.13, p=.04	0.37%	-0.15, p=.02	-10%
Glycerophosphoethanolamines: C34:3 PE plasmalogen	-0.13, p=.03	-0.14, p=.02	-6.3%	-0.13, p=.03	0.86%	-0.18, p=.003	-35%	-0.08, p=.21	-15%
C36:4 PE	0.13, p=.03	0.11, p=.06	17%	0.10, p=.10	24%	0.11, p=.07	16%	0.07, p=.31	43%
C44:13 PE plasmalogen	-0.12, p=.049	-0.13, p=.03	-4.4%	-0.10, p=.10	16%	-0.12, p=.04	-5.3%	-0.07, p=.29	23%
Ceramides: C24:1 ceramide (d18:1)	0.13, p=.03	0.11, p=.07	4.1%	0.13, p=.03	1.3%	0.14, p=.02	-5.4%	0.12, p=.06	-6.9%
Glycerophosphocholines: C20:5 LPC	-0.12, p=.04	-0.13, p=.03	0.69%	-0.10, p=.11	23%	-0.11, p=.07	13%	-0.06, p=.36	40%
Bile acids, alcohols/derivatives: Glycodeoxycholate	-0.12, p=.04	-0.12, p=.04	0.72%	-0.11, p=.06	7.3%	-0.11, p=.06	8.2%	-0.13, p=.045	-13%

Log-transformed and standardized Metabolites	Age/site-adjusted correlation with SAVE (N=287)	F) Adjusting for creatinine (n=284[a])		G) Adjusting for prevalent diseases[b] (N=287)		H) Adjusting for medications[c] (N=287)		I) Adjusting for multiple variables[d] (n=257[a])	
		Correlation	% attenuation	Correlation	% attenuation	Correlation	% attenuation	Correlation	% attenuation
Triradylcglycerols:									
C54:10 TAG	-0.12, p=.046	-0.12, p=.05	-5.7%	-0.13, p=.02	-14%	-0.13, p=.02	-13%	-0.16, p=.01	-23%
Steroid esters:									
C18:2 CE	-0.13, p=.03	-0.15, p=.01	-4.6%	-0.11, p=.07	17%	-0.12, p=.04	6.5%	-0.09, p=.17	22%
**Organoheterocyclic compounds:**									
Indolyl carboxylic acids/derivatives:									
Tryptophan	-0.21, p=.0005	-0.17, p=.004	16%	-0.20, p=.0007	1.8%	-0.19, p=.001	6.8%	-0.18, p=.005	15%
Pyridinecarboxylic acids/derivatives:									
1-methylnicotinamide	-0.15, p=.01	-0.17, p=.004	-12%	-0.16, p=.009	-3.4%	-0.17, p=.004	-13%	-0.18, p=.004	-25%
Purines and purine derivatives:									
Urate	0.13, p=.03	0.09, p=.13	32%	0.13, p=.03	-1.4%	0.12, p=.05	10%	0.11, p=.09	21%
Tryptamines and derivatives:									
5-hydroxytryptophan	-0.12, p=.04	-0.07, p=.24	41%	-0.12, p=.047	0.52%	-0.09, p=.11	21%	-0.10, p=.13	31%
**Benzenoids:**									
1-hydroxy-2-unsubstituted benzenoids:									
Hydroxyphenylacetate	0.15, p=.01	0.08, p=.16	41%	0.10, p=.08	32%	0.08, p=.16	45%	-0.004, p=.95	103%
Benzoic acids and derivatives:									
Salicylurate	0.12, p=.04	0.08, p=.21	36%	0.05, p=.40	59%	0.04, p=.51	68%	-0.02, p=.75	118%
Phenylacetic acids:									
Homogentisate	0.12, p=.04	0.08, p=.16	22%	0.08, p=.16	32%	0.09, p=.12	24%	0.08, p=.19	45%
**Organic nitrogen compounds:**									
Amines:									
Putrescine	0.15, p=.01	0.11, p=.06	19%	0.15, p=.009	-2.6%	0.14, p=.02	6.5%	0.11, p=.09	16%
Aminoxides:									
Trimethylamine-N-oxide	0.13, p=.03	0.07, p=.25	47%	0.10, p=.10	24%	0.07, p=.27	49%	0.02, p=.73	80%
**Nucleosides/nucleotides/analogues:**									
Sub class not available:									
Inosine	0.14, p=.02	0.12, p=.046	11%	0.13, p=.03	3.3%	0.15, p=.01	-11%	0.12, p=.06	16%
**Organic oxygen compounds:**									
Carbohydrates/carbohydrate conjugates:									
Glucuronate	0.21, p=.0003	0.17, p=.004	25%	0.18, p=.002	15%	0.15, p=.01	30%	0.12, p=.07	49%

Percent attenuation=100*(r_1-r_2)/r_1; where r_1=correlation coefficient between SAVE scores and a metabolite adjusting for age and study site, r_2=correlation coefficient after further adjustments

[a]For the more commonly measured variables that were not measured in all 287 participants: the correlation coefficient that was further adjusted for that variable was compared to the age- and site-adjusted correlation coefficient restricted to the same sample size to calculate percent attenuation.

[b]Prevalent diseases: cardiovascular diseases, hypertension, diabetes, cancer, peripheral arterial disease, osteoarthritis, depression, and pulmonary diseases

[c]Medications: anti-hypertensives, antilipemics, medications for diabetes, and medications for pulmonary diseases

[d]Current smoking status, body mass index, appendicular lean mass, % fat, daily protein intake, interleukin-6, C-reactive protein, creatinine, cardiovascular disease, cancer, peripheral arterial disease, osteoarthritis, depression, number of prescription medications, anti-hypertensives, antilipemics, medications for diabetes, medications for pulmonary diseases

Shading: blue indicates negative attenuation≤-10% (stronger association after adjustment) and red indicates positive attenuation≥10% (weaker association after adjustment)

Table S3. Metabolites detected among overnight-fasting plasma samples from 287 Health ABC black men by the three profiling platforms

Method	Metabolites (M=334)
Lipid profiling method (C8-positive; m=187)	C14:0 LPC, C16:0 LPC, C16:1 LPC, C18:0 LPC, C18:1 LPC, C18:2 LPC, C20:3 LPC, C20:4 LPC, C20:5 LPC, C22:6 LPC, C30:0 PC, C30:1 PC, C32:0 PC, C32:1 PC, C32:2 PC, C34:0 PC, C34:1 PC, C34:1 PC plasmalogen-A, C34:2 PC, C34:2 PC plasmalogen, C34:3 PC, C34:3 PC plasmalogen, C34:4 PC, C36:0 PC, C36:1 PC, C36:1 PC plasmalogen, C36:2 PC, C36:2 PC plasmalogen, C36:3 PC, C36:3 PC plasmalogen, C36:4 PC plasmalogen, C36:4 PC-A, C36:4 PC-B, C36:5 PC plasmalogen-A, C36:5 PC plasmalogen-B, C38:2 PC, C38:3 PC, C38:4 PC, C38:4 PC plasmalogen, C38:6 PC, C38:6 PC plasmalogen, C38:7 PC plasmalogen, C40:10 PC, C40:6 PC, C40:7 PC plasmalogen, C40:9 PC, C16:0 LPE, C18:0 LPE, C18:1 LPE, C18:2 LPE, C20:4 LPE, C22:6 LPE, C34:0 PE, C34:2 PE, C34:2 PE plasmalogen, C34:3 PE plasmalogen, C36:0 PE, C36:1 PE, C36:2 PE, C36:2 PE plasmalogen, C36:3 PE, C36:3 PE plasmalogen, C36:4 PE, C36:4 PE plasmalogen, C36:5 PE plasmalogen, C38:2 PE, C38:3 PE plasmalogen, C38:4 PE, C38:5 PE, C38:5 PE plasmalogen, C38:6 PE, C38:6 PE plasmalogen, C38:7 PE plasmalogen, C40:6 PE, C40:7 PE plasmalogen, C42:11 PE plasmalogen, C44:13 PE plasmalogen, C32:1 PI, C32:2 PI, C34:0 PI, C34:2 PI, C38:4 PI, C34:0 PS, C42:0 TAG, C44:0 TAG, C44:1 TAG, C44:2 TAG, C46:0 TAG, C46:1 TAG, C46:2 TAG, C46:3 TAG, C46:4 TAG, C48:0 TAG, C48:1 TAG, C48:2 TAG, C48:3 TAG, C48:4 TAG, C48:5 TAG, C50:0 TAG, C50:1 TAG, C50:2 TAG, C50:3 TAG, C50:4 TAG, C50:5 TAG, C50:6 TAG, C52:0 TAG, C52:1 TAG, C52:2 TAG, C52:3 TAG, C52:4 TAG, C52:5 TAG, C52:6 TAG, C52:7 TAG, C54:1 TAG, C54:10 TAG, C54:2 TAG, C54:3 TAG, C54:4 TAG, C54:5 TAG, C54:6 TAG, C54:7 TAG, C54:8 TAG, C56:1 TAG, C56:10 TAG, C56:2 TAG, C56:3 TAG, C56:4 TAG, C56:5 TAG, C56:6 TAG, C56:7 TAG, C56:8 TAG, C56:9 TAG, C58:10 TAG, C58:11 TAG, C58:6 TAG, C58:7 TAG, C58:8 TAG, C58:9 TAG, C30:0 DAG, C32:0 DAG, C32:1 DAG, C32:2 DAG, C34:0 DAG, C34:1 DAG, C36:0 DAG, C36:1 DAG, C36:2 DAG, C38:4 DAG, C38:5 DAG, C14:1 MAG, C16:1 MAG, C18:0 MAG, C22:1 MAG, C34:2 DAG, C34:3 DAG, C36:3 DAG, C36:4 DAG, C14:0 SM, C16:0 SM, C18:0 SM, C18:1 SM, C20:0 SM, C22:0 SM, C22:1 SM, C24:0 SM, C24:1 SM, C16:1 SM, C18:2 SM, C16:0 ceramide (d18:1), C22:0 ceramide (d18:1), C24:0 ceramide (d18:1), C24:1 ceramide (d18:1), C16:0 CE, C16:1 CE, C18:0 CE, C18:1 CE, C18:2 CE, C18:3 CE, C20:3 CE, C20:4 CE, C20:5 CE, C22:4 CE, C22:5 CE, C22:6 CE, C36:1 PS plasmalogen, C36:2 PS plasmalogen, sphingosine
Positive ion mode MS detection (HILIC-positive; m=87)	Alpha-glycerophosphocholine, C5:1 carnitine, C5 carnitine, C10 carnitine, C12 carnitine, C14 carnitine, C14:1 carnitine, C16 carnitine, C18 carnitine, C18:1 carnitine, C18:2 carnitine, C2 carnitine, C26 carnitine, C3 carnitine, C4 carnitine, C6 carnitine, C8 carnitine, mevalonic acid, 5-aminolevulinic acid, acetylglycine, Asymmetric dimethylarginine (ADMA), alanine, aminoisobutyric acid, arginine, asparagine, beta-alanine, betaine, citrulline, creatine, creatinine, dimethylglycine, Gamma-Aminobutyric acid (GABA), glutamate, glutamine, histidine, hydroxyproline, isoleucine, leucine, lysine, methionine, methionine sulfoxide, N-Monomethyl-arginine (NMMA), ornithine, phenylalanine, pipecolic acid, proline, pyroglutamic acid, sarcosine, Symmetric dimethylarginine (SDMA), serine, threonine, thyroxine, tyrosine, valine, C4-OH carnitine, C12:1 carnitine, C16-OH carnitine, C18:1-OH carnitine, C3-DC-CH3 carnitine, C5-DC carnitine, C7 carnitine, C9 carnitine, taurine, phosphoethanolamine, N-carbamoyl-beta-alanine, 5-hydroxytryptophan, 1-methylnicotinamide, allantoin, cytosine, tryptophan, serotonin, niacinamide, cotinine, kynurenic acid, adenosine, xanthosine, acetylcholine, 1-methylhistamine, histamine, putrescine, trimethylamine-N-oxide, carnitine, choline, 3-hydroxyanthranilic acid, C10:2 carnitine, C14:2 carnitine, C20:4 carnitine
Negative ion mode MS detection (HILIC-negative; m=60)	Alpha-glycerophosphate, adipate, Guanosine monophosphate (GMP), 3-methyladipate/pimelate, chenodeoxycholate/deoxycholate, glycocholate, glycodeoxycholate/glycochenodeoxycholate, 2-aminoadipate, cystathionine, phosphocreatine, beta-hydroxybutyrate, malate, alpha-hydroxybutyrate, lactate, 2-hydroxyglutarate, fumarate/maleate, methylmalonate, oxalate, succinate, aconitate, citrate, isocitrate, pyruvate, alpha-ketoglutarate, indoxylsulfate, uracil, hypoxanthine, urate, xanthine, indole-3-propionate, quinolinate, 4-pyridoxate, xanthurenate, 2-phosphoglycerate, inositol, pantothenate, fructose/glucose/galactose, glucoronate, hexose monophosphate, lactose, sorbitol, sucrose, kynurenine, Malondialdehyde (MDA), inosine, cAMP, Adenosine diphosphate (ADP), Adenosine monophosphate (AMP), Guanosine diphosphate (GDP), Inosinic acid (IMP), uridine, Uridine diphosphate (UDP)-glucose, uridine 5'-diphosphate (UDP), 4-hydroxymandelate, benzoate, gentisate, hippurate, salicylurate, homogentisate, hydroxyphenylacetate,

Table S4. Taxonomy classification based on the Human Metabolome Database of 334 metabolites detected in overnight-fasting plasma from 287 Health ABC black men

HMDB Taxonomy super class	HMDB Taxonomy class	HMDB Taxonomy sub class
Lipids and lipid-like molecules (m=211)	Glycerophospholipids (m=85)	Glycerophosphocholines (m=47)
		Glycerophosphoethanolamines (m=31)
		Glycerophosphoinositols (m=5)
		Glycerophosphoserines (m=1)
		Glycerophosphates (m=1)
	Glycerolipids (m=70)	Triradylcglycerols (m=55)
		Diradylglycerols (m=11)
		Monoradylglycerols (m=4)
	Fatty acyls (m=24)	Fatty acid esters (m=16)
		Lineolic acids and derivatives (m=4)
		Fatty acids and conjugates (m=3)
		Eicosanoids (m=1)
	Sphingolipids (m=15)	Phosphosphingolipids (m=9)
		Ceramides (m=4)
		Not available (m=2)
	Steroids and steroid derivatives (m=15)	Steroid esters (m=12)
		Bile acids, alcohols and derivatives
	Not available (m=2)	Not available (m=2)
Organic acids and derivatives (m=65)	Carboxylic acids and derivatives (m=46)	Amino acids, peptides, and analogues (m=39)
		Dicarboxylic acids and derivatives (m=4)
		Tricarboxylic acids and derivatives (m=3)
	Hydroxy acids and derivatives (m=13)	Beta hydroxy acids and derivatives (m=10)
		Alpha hydroxy acids and derivatives (m=2)
		Short-chain hydroxy acids and derivatives (m=1)
	Keto acids and derivatives (m=2)	Alpha-keto acids and derivatives (m=1)
		Gamma-keto acids and derivatives (m=1)
	Organic sulfuric acids and derivatives (m=2)	Arylsulfates (m=1)
		Organosulfonic acids and derivatives (m=1)
	Organic phosphoric acids and derivatives (m=1)	Phosphate esters (m=1)
	Organic carbonic acids and derivatives (m=1)	Ureas (m=1)
Organoheterocyclic compounds (m=17)	Pyridines and derivatives (m=5)	Pyridinecarboxylic acids and derivatives (m=4)
		Pyrrolidinylpyridines (m=1)
	Imidazopyrimidines (m=3)	Purines and purine derivatives (m=3)
	Indoles and derivatives (m=2)	Indolyl carboxylic acids and derivatives
	Diazines (m=2)	Pyrimidines and pyrimidine derivatives
	Quinolines and derivatives (m=2)	Quinoline carboxylic acids
	Indoles and derivatives (m=2)	Tryptamines and derivatives
	Azoles (m=1)	Imidazoles (m=1)
Organic oxygen compounds (m=11)	Organooxygen compounds (m=11)	Carbohydrates and carbohydrate conjugates (m=6)
		Alcohols and polyols (m=2)
		Carbonyl compounds (m=2)
		Carbohydrates and carbohydrate conjugates (m=1)
Nucleosides, nucleotides, and analogues (m=11)	Purine nucleosides (m=7)	Purine ribonucleotides (m=4)
		Cyclic purine nucleotides (m=1)
		Not available (m=2)
	Pyrimidine nucleosides (m=4)	Pyrimidine nucleotide sugars (m=2)
		Pyrimidine ribonucleotides (m=1)
		Not available (m=1)
Organic nitrogen compounds (m=8)	Organonitrogen compounds (m=8)	Amines (m=4)
		Quaternary ammonium salts (m=3)
		Aminoxides (m=1)
Benzenoids (m=8)	Benzene and substituted derivatives (m=5)	Benzoic acids and derivatives (m=4)
		Phenylacetic acids (m=1)
	Benzene and substituted derivatives (m=1)	Benzoic acids and derivatives (m=1)
	Phenols (m=2)	1-hydroxy-2-unsubstituted benzenoids (m=2)
Not available (m=3)	Not available (m=3)	Not available (m=3)

HMDB=Human Metabolome Database (Reference: Wishart, D.S. et al. HMDB: the human metabolome database. Nucleic acids research. 2007, 35(suppl_1), D521-D526.)
